# Attraction Effects for Verbal Gender and Number Are Similar but Not Identical: Self-Paced Reading Evidence From Modern Standard Arabic

**DOI:** 10.3389/fpsyg.2020.586464

**Published:** 2021-01-21

**Authors:** Matthew A. Tucker, Ali Idrissi, Diogo Almeida

**Affiliations:** ^1^Language, Mind and Brain Lab, Division of Science, Psychology Program, New York University Abu Dhabi, Abu Dhabi, United Arab Emirates; ^2^Amazon.com, Inc., Cambridge, MA, United States; ^3^The Neurocognition of Language Lab, Department of English Literature and Linguistics, Qatar University, Doha, Qatar

**Keywords:** Arabic, agreement, agreement attraction, self-paced reading, verbal gender, verbal number, phi-features, meta-analysis

## Abstract

Previous work on the comprehension of agreement has shown that incorrectly inflected verbs do not trigger responses typically seen with fully ungrammatical verbs when the preceding sentential context furnishes a possibly matching distractor noun (i.e., agreement attraction). We report eight studies, three being direct replications, designed to assess the degree of similarity of these errors in the comprehension of subject-verb agreement along the dimensions of grammatical gender and number in Modern Standard Arabic. A meta-analysis of the results demonstrate the presence of agreement attraction effects in reading comprehension for gender and number on verbs. Moreover, the meta-analysis demonstrates that these two features do not behave identically: gender effects are larger and occur later relative to number attraction effects. These results challenge models of agreement that predict agreement features to be equipotent and show that real-time models of agreement require modifications in the form of cue-weighting in order to account for these differential results.

## 1. Introduction

Human language contains many relationships between words which can obtain at a distance. Subject-verb agreement as in (1) is one such relationship:

**Table:** 

(1)	a.	**The fencers** ***are*** divided about their strategy for the World Championships.
	b.	**The fencers** on the French National Team that won a major award last year by beating the Italian team in a very hotly contested and important match ***are*** deeply divided about the best strategy for the World Championships.

In the specific case of (1a), the choice of *the fencers* conditions the subsequent choice of *are* in production or the expectation of a plural verb in comprehension. Subject-verb agreement is particularly important in the study of language and its relationship with the performance systems since it not only involves the very basic building blocks of a clause but also because it is a relationship that can obtain at an unbounded serial distance. Subjects can theoretically be separated from their verbs by an infinite amount of material and yet still require proper agreement—see (1b). This basic fact underscores an important property of the syntax of human languages: despite their linear externalization, sentences are internally organized in a hierarchical, and not serial, fashion.

Therefore, from the perspective of real-time language production and comprehension, coping with potentially unbounded dependencies, such as subject-verb agreement requires attention to the encoding, maintaining, and retrieving of linguistic units from working memory, as well as the monitoring process that oversees whether the correct relationship between the subject and the verb has been completed. It is remarkable, then, that subject-verb agreement errors are not only sometimes observed both in language production (Bock and Miller, 1991), and comprehension (Pearlmutter et al., 1999; Wagers et al., 2009), but that they also seem to be at least partially systematic. Known as agreement attraction, a particularly well-studied subset of these errors are commonly seen when a subject co-occurs with a non-subject argument that appears to be the target of the erroneous number agreement, as in the example in (2) from Dillon et al. (2013)^1^:

(2) *The executive* who oversaw **the middle managers** apparently ***were*** dishonest about the company's profits.

The characteristic property of this phenomenon is the illusion of acceptability for *prima facie* unacceptable agreement violations—despite the fact that the plural *were* is ungrammatical in (2), many speakers occasionally both accept and produce such utterances. In production studies, such as Bock and Miller (1991) or Franck et al. (2002), these errors surface as incorrect verb productions, whereas in comprehension studies, such as Pearlmutter et al. (1999) or Tanner et al. (2014), these errors surface as the absence of behavioral or electrophysiological responses typically associated with the perception of ungrammaticality.

Because they represent a systematic exception to the idea that online language processing follows grammatical rules during the production and comprehension of dependencies, many researchers interpret attraction violations as a window into either the processes via which long-distance dependencies are (sometimes erroneously) encoded in real-time language comprehension (e.g., Bock and Eberhard, 1993; Nicol et al., 1997; Pearlmutter et al., 1999; Eberhard et al., 2005; Franck et al., 2008), or the processes by which encoded linguistic structures are manipulated and searched in memory in the course of language comprehension (e.g., the cue-based memory retrieval models assumed by Lewis and Vasishth, 2005; Badecker and Lewis, 2007; Badecker and Kuminiak, 2007; Wagers et al., 2009; Dillon et al., 2013).

### 1.1. Assumptions About Agreement Features

Despite its potential to shed light on the relationship between linguistic representations and their online processing, research on agreement attraction in subject-verb dependencies has focused primarily on the process of number agreement. The existing data on agreement attraction involving features other than number are sparse and equivocal. For example, the process of subject-verb gender agreement has elicited conflicting attraction results in production studies in Slavic (Lorimor et al., 2008; Badecker and Kuminiak, 2007). In comprehension, Slioussar and Malko (2016) is the only study documenting attraction effects in subject-verb gender agreement (see Villata and Franck, 2020 for recent evidence on *object-verb* gender agreement in French in grammatical sentences). However, none of these studies have directly addressed the comparative magnitude of these effects with respect to attraction errors in the better studied case of subject-verb number agreement^2^.

This is an important gap in the literature, as most linguistic and psycholinguistic theories of agreement naturally posit *equivalence* across agreement features. Namely, linguistic theory generally takes person, number, and gender features to be *equipotent* in agreement phenomena (e.g., Pollock, 1989; Chomsky, 1995; Preminger, 2011; though see Béjar, 2003; Béjar and Rezac, 2009 for a different approach). This assumption is generally mirrored in psycholinguistic theories which take retrieval cues as isomorphic to linguistic features, therefore predicting *equivalence* in attraction effects for different agreement features. For instance, misrepresentation theories (e.g., Bock and Eberhard, 1993; Nicol et al., 1997; Pearlmutter et al., 1999; Eberhard et al., 2005; Franck et al., 2008) attribute agreement attraction to normal mechanisms of feature spreading, and thus differences in attraction strength for different features are only predicted if representational considerations constrain spreading, overwriting, or copying. Cue-based memory search models, on the other hand, posit that cues are typically treated equally by the retrieval system and at least partially depend on linguistic features (e.g., Lewis and Vasishth, 2005; Badecker and Lewis, 2007; Badecker and Kuminiak, 2007; Wagers et al., 2009; Dillon et al., 2013). Any observed difference between how different agreement cues are processed would necessitate positing a more complex view of these cues or how they are weighed or retrieved within the memory system. It is therefore crucially important to determine whether this basic assumption—namely that all agreement features are equipotent—which is shared by the two most popular families of theories of agreement errors as well as by the representational linguistic theories they explicitly or implicitly assume, is, in fact, supported by the evidence.

In an effort to systematically document the ways in which subject-verb agreement processes are similar and the ways in which they are different depending on the agreement feature of interest, we report a series of eight comprehension studies in Modern Standard Arabic (MSA) in which we directly compare the process of subject-verb gender agreement with the process of subject-verb number agreement. MSA provides several important *desiderata* for studies of verbal gender (Ryding, 2005): (1) the presence of verbal gender agreement on all verbs in the language; (2) the appearance of gender marking on nominals independent of case morphology, allowing the examination of gender independently of the influence of case; (3) a demonstrated number attraction effect in comprehension against which to compare results from gender (Tucker et al., 2015); and (4) a close typological relationship to Hebrew, a language which has been the focus of some production work (Deutsch and Dank, 2009, 2011; Dank and Deutsch, 2010) and in which gender and number emerge as *not equivalent* in the production of attraction errors, at least as far as noun-adjective agreement is concerned.

### 1.2. Common Structure of Experiments

The eight experiments reported here draw on minimal variations of a common experimental structure: the presence or absence of attraction effects upon the presentation of preverbal subject relative clause modifiers in Modern Standard Arabic in a self-paced moving window paradigm (Just et al., 1982). The experimental stimuli for each study thus involve sentences of the structure *NP1—Complementizer—[Verb—NP2—Adverb]—****Target Verb***—*Continuation*, where NP1 is the grammatically accessible subject and NP2 the attractor NP for agreement realized on the target verb. An attraction effect in a self-paced reading presentation of a sentence of this structure is therefore manifested as facilitated reading times to attraction configuration relative to ungrammatical controls at the *Target Verb* region (Pearlmutter et al., 1999; Wagers et al., 2009; Dillon et al., 2013).

All experiments employ subject relative clauses (see e.g., Bock and Miller, 1991; Dillon et al., 2013) modifying a sentence-initial subject. Since number agreement attraction in this configuration has already been studied in MSA (Tucker et al., 2015), this facilitates the direct comparison of the reaction time profiles of grammatical number and gender processing. Moreover, Wagers et al. (2009) have shown that spillover effects in agreement attraction studies can inadvertently impact measurements at critical verbs when the immediately previous region is manipulated experimentally (see also Jäger et al., 2017)—an adverb placed at the end of the relative-clause in our stimuli obviates this concern. An example sentence from the stimuli for Experiment 1 which conforms to these design features is shown in (3):


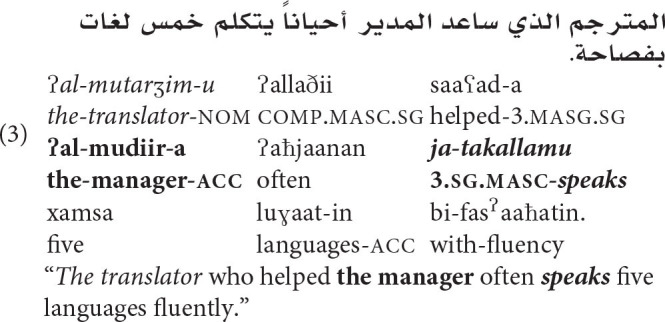


In addition to the requirements discussed above, several other constraints were also placed on the creation of stimuli sentences: Firstly, the relative clause verbs were chosen such that they either took a bare NP complement or a PP complement headed by a preposition which is orthographically encliticized to the relative clause direct object in order to ensure that all stimuli had the same number of words up to the main clause target verb. Secondly, Arabic has two distinct morphological tenses which are marked on verbs in part by distinct agreement affixes (Ryding, 2005, p. 439–444). In order to abstract away from the individual contributions of distinct tense/agreement affixes, the main clause target verbs were counterbalanced for the two tenses, perfect (e.g., تكلم/*takallam*, “he spoke”) and imperfect (e.g., يتكلم/*ja-takallam*, “he speaks”).

Finally, in order to facilitate cross-experimental comparisons, all eight studies were designed to contain a shared sub-design involving two manipulations: (i) Match (does the attractor have the same agreement cues as the subject?), which has either the value *yes* or *no* and (ii) Grammaticality (does the subject have the same agreement cues as the verb?), which can be either the value *grammatical* or *ungrammatical*. An example of the coding scheme for this shared manipulation is shown for the English translation of (3) in 4 below:

**Table:** 

	The *translator* who helped…	
(4)	a.	the president often **speaks**… Match/**Gram**
	b.	the president often **speak**… Match/**Ungram**
	c.	the presidents often **speaks**… NoMatch/**Gram**
	d.	the presidents often **speak**… NoMatch/**Ungram**

### 1.3. Statistical Analysis

As discussed above, all experiments are based on nearly identical stimuli and design. This allows us to perform a *meta-analysis* of the cumulative evidence presented here. In order to facilitate such a meta-analysis, we report the experimental results using estimation of effect sizes (in raw RT measurements), and 95% confidence intervals (CI; all calculated via the BCa Bootstrap with 2000 replications per estimate; cf. Efron, 1987; Kirby and Gerlanc, 2013).

In eschewing the presentation of results in terms of Null Hypothesis Significance Tests (NHST) and their associated *p*-values, we follow the advice of a number of statistical reformers (Cohen, 1994; Cumming, 2014 for review), including the American Statistical Association and the Task force on Statistical Inference of the American Psychological Association (Wasserstein and Lazar, 2016; Wilkinson, 1999). *P*-values are easily and often misconstrued (Cohen, 1994; Greenland et al., 2016; Gigerenzer, 2004; Haller and Krauss, 2002), promote unhelpful dichotomous thinking about the results (Cumming, 2014; Wasserstein and Lazar, 2016) and do not easily support cumulative weighing of the evidence (for instance, it is unclear what conclusions follow from replications that fail to reach *p* < 0.05; cf. Maxwell et al., 2015; Hedges and Schauer, 2019).

CIs are ultimately based on the same underlying statistical theory of NHST, and are not impervious to misinterpretation (Greenland et al., 2016), but they can assist in the judicious evaluation of data by explicitly providing information about the uncertainty surrounding the measurement of interest and the range of values that may or may not be compatible with it beyond the null hypothesis (Cohen, 1994; Cumming, 2014; Hoekstra et al., 2012). CIs can also inform future research due to their ability to function as *prediction intervals*: a 95% CI provides an 83% prediction interval for replications with identical sample sizes (Cumming, 2008 cf. Spence and Stanley, 2016 for unequal sample sizes). Finally, CIs also allow interpretations that go beyond their original statistical framework when used as proxies for other measures: CIs closely approximate Bayesian Credible Intervals (CrI) when *non-informative* priors are used (Albers, 2018), and also the *number needed to disturb* (NNTD; Gorard, 2019), a measure of the robustness of the data that quantifies the amount of counterfactual variation that would be necessary to disturb a given finding.

It is nonetheless possible to connect the results of a 95% CI to a statement about statistical significance based on *p* < 0.05. If the 95% CI for a particular effect excludes 0, then *p* < 0.05. Comparing independent CIs to each other, however, requires a bit more caution, as independent CIs can sometime overlap by as much as 58% and still clear the traditional *p* < 0.05 threshold (Cumming and Finch, 2005; Krzywinski and Altman, 2013). In order to address this issue, Cumming and Finch (2005) propose the following rule-of-thumb: if the two CIs overlap by a proportion of up to 50% of the average width of the two CIs, then *p* ≤ 0.05. If two independent 95% CIs do not overlap, then *p* ≤ 0.01 (Cumming and Finch, 2005; Krzywinski and Altman, 2013). For the reader's convenience, we will also highlight CIs that include 0 up to ±1 ms as potentially suggestive evidence of an experimental effect in all the result tables, an informal analogy with the *marginally significant* range sometimes used in traditional NHST. In order to contribute to the effort of increasing reproducibility and replicability of findings in the cognitive sciences, the full data, as well as the analysis scripts, are publicly available at https://figshare.com/projects/Attraction_Effects_for_Verbal_Gender_and_Number_Are_Similar_but_Not_Identical_Self-Paced_Reading_Evidence_from_Modern_Standard_Arabic/18823.

In all experiments, we have two basic effects of interest, (i) whether grammaticality affects reading times (the “grammaticality effect”) and (ii) whether ungrammatical sentences with partially matching distractor NPs have erroneous facilitation (the “attraction effect”). These are estimated within-subjects, and their 95% CI is then calculated via BCa bootstrap (Efron, 1987; Kirby and Gerlanc, 2013). In each experiment, the grammaticality effect is quantified for each participant by summing their average reaction times for the ungrammatical sentences (i.e., those sentences in which the subject mismatches the verb in either number or gender) and subtracting the sum of their average reaction times for the grammatical sentences from it. The attraction effect is quantified separately for grammatical and ungrammatical sentences, since prior work has noticed that attraction has a tendency to occur in *ungrammatical* sentences alone (the grammaticality asymmetry mentioned by Wagers et al., 2009 and Jäger et al., 2017. However, this effect may not obtain for children; see Adani et al., 2012 and Belletti et al., 2012). In our coding scheme, the attraction effect is a subtraction of the average reading time from NoMatch condition from the Match condition, within each level of Grammaticality (Dillon et al., 2013).

### 1.4. Assessing Equivalency Between Number and Gender

We identify five distinct ways in which gender and number could be equivalent in regards to comprehension attraction effects:

**Table:** 

(5)
	a.	Existence: Do both features participate in attraction?
	b.	Size: Do both features yield similar attraction effect magnitudes?
	c.	Grammatical Asymmetry: Do both features participate in asymmetric attraction effects based on grammaticality of the verb?
	d.	Markedness Asymmetry: Do both features participate in asymmetric attraction effects based on markedness of the agreeing elements?
	e.	Timing: Do both features exhibit attraction effects with the same time-course?

Out of all these criteria, special attention in all experiments will be given to whether grammatical asymmetries in attraction effects are observed. As discussed by Wagers et al. (2009), if attraction is a product of the *process* of resolving agreement dependencies, then we do not expect to find attraction profiles in grammatical sentences, a prediction that can be made consistent with cue-based memory retrieval models. However, if attraction is instead due to *fallibility* in the representation of agreement features, we expect to find no differential attraction effect owing to the grammatical status of the sentence. Therefore, the presence or absence of grammatical asymmetries in attraction effect profiles offers selective support to either misrepresentation theories or cue-based retrieval models. The size, markedness asymmetry, and timing dimensions are considered with respect to the equivalency of agreement features discussed in the preceding section.

Experiment 1 assesses whether verbal gender agreement is subject to attraction effects in Modern Standard Arabic. Experiment 2 and its direct replication (named 2A and 2B, respectively) assess whether gender attraction is replicable and whether it displays a markedness asymmetry. Experiment 3 then turns to number attraction by replicating and extending the original results of Tucker et al. (2015). Experiment 4 and its direct replication (4A and 4B, respectively) then assess whether number agreement attraction displays markedness asymmetry. Finally, Experiment 5 and its replication (5A and 5B, respectively) provide a within-subjects comparison of gender and number attraction. We then conclude with a meta-analysis of all eight studies which also includes the data from Tucker et al. (2015).

## 2. Experiment 1: Gender Attraction

Experiment 1 was designed to assess whether attraction effects in the processing of subject-verb gender agreement obtains in MSA comprehension. Given the prevailing linguistic and psycholinguistic conceptions of agreement, one expects to find that attraction effects should be possible for gender. In the formal syntactic literature, agreement is often taken to be a uniform process which simultaneously encompasses the features of gender, number, and person (Pollock, 1989; Chomsky, 1995, 2000, 2001). Furthermore, both misrepresentation models and cue-based retrieval models require added mechanics to differentiate cues for number and gender, meaning that gender should, if isolated properly, behave similarly to number in comprehension.

### 2.1. Participants

Participants were 104 native speakers of Arabic from the United Arab Emirates University (UAEU) student body with no history of language disorders and self-assessed proficiency with MSA (104 females; mean age 20.4 years).^3^ All participants provided informed consent and were compensated monetarily for their time in all experiments in this study. This and all other studies reported here were approved by the NYU Abu Dhabi Institutional Review Board and the UAEU Ethics Committee.

### 2.2. Materials

In order to assess the possibility of gender attraction in MSA, a set of 48 sentences meeting the criteria outlined in the introduction were constructed. With the NP subject (NP1) and attractor (NP2), nouns were chosen which had a masculine stem which could be made feminine solely by addition of the feminine singular nominal suffix /-a/ (orthographic ــة)—in MSA these are easiest to find in the domain of nouns which denote human occupations. While MSA does have nouns which are feminine without the presence of this suffix, restriction to these nouns was employed in order to abstract away from possible differences in the processing of nominal gender owing to whether or not the feminine gender was an inherent property of the stem vs. the contribution of an overt suffix (Sicuro Corrêa et al., 2004). Moreover, the choice of an overtly suffixing feminine allows a straightforward comparison between the processing of gender in MSA and suffixal plural morphology in other languages. A complete list of stimuli for this experiment appears in the Supplementary Material.

For each experimental sentence, four variants were constructed by systematically varying the grammatical gender of the attractor (NP2) and the main clause verb (target verb). These manipulations are coded as Match and Grammaticality as described above. Note that in this design, NoMatch conditions are conditions with feminine attractors, since all subjects are masculine. Both relevant NPs remained in the singular throughout the experiment to assess the effect of gender alone. This resulted in four experimental conditions per stimulus; a complete set of four such sentences appears in Table 1.

**Table 1:** A complete item set for one stimulus in Experiment 1.

**Condition**	**NP1**	**Comp**	**RCV**	**NP2**	**Adv**	**V**	**Continuation**
	**R1**	**R2**	**R3**	**R4**	**R5**	**R6**	**R7–R_n_**
MATCH/GRAM	المترجم	الذي	ساعد	المدير	أحياناً	يتكلم	.خمس لغات بفصاحة
	The translator (MASC)	who	helped	the manager (MASC)	often	speaks (MASC)	five languages fluently.
MATCH/UNGRAM	المترجم	الذي	ساعد	المدير	أحياناً	تتكلم	.خمس لغات بفصاحة
	The translator (MASC)	who	helped	the manager (MASC)	often	speaks (FEM)	five languages fluently.
MATCH/GRAM	المترجم	الذي	ساعد	المديرة	أحياناً	يتكلم	.خمس لغات بفصاحة
	The translator (MASC)	who	helped	the manager (FEM)	often	speaks (MASC)	five languages fluently.
MATCH/GRAM	المترجم	الذي	ساعد	المديرة	أحياناً	تتكلم	.خمس لغات بفصاحة
	The translator (MASC)	who	helped	the manager (FEM)	often	speaks (FEM)	five languages fluently.

The 48 sets of four sentences were distributed across four lists in a Latin Square design after being combined with 144 grammatical fillers of similar length for a 3:1 filler-to-item ratio. None of the fillers included the relative clause construction used in the experimental stimuli or any construction which drew attention to meaningful alternations in verbal agreement. In the final version of each list, only the experimental sentences contained ungrammaticalities, with 12.5% of the sentences in each list ungrammatical.

### 2.3. Procedure

Subjects were seated comfortably up to eight at a time at a table in a quiet room in front of Apple iMac computers running Windows 7 natively via a Boot Camp partition on which the experimental software had been pre-loaded. Sentences were presented using the Linger software (Rhode, 2003) in a self-paced word-by-word moving window paradigm (Just et al., 1982). Each trial began with the display of a screen containing the sentence masked by dashes (including spaces and punctuation). Each time the participant pressed the space bar, a single word was revealed and the previous word re-masked with no look-back allowed. All items were presented in the Courier New Arabic font in 28pt bold type. A yes/no comprehension question followed each sentence, appearing on the screen all at once. Comprehension questions were designed in such a way that the answer could be provided independent of experimental manipulations—no questions asked about the attractor NP or the main clause verb. None of our comprehension questions required lexical elaboration of the item or difficult semantic processing. A majority of the comprehension questions asked about the relative clause verb or the post-critical region continuation. As an example, the item *The student who saw the professor(s) yesterday studied electrical engineering at the university* was followed by the question *Did the student study electrical engineering?* Participants responded via a dual Arabic/English keyboard where the “f/ب” key was used for “yes (نعم)” and the “j/ت” key used for “no (لا).” Onscreen feedback was provided for both correct and incorrect answers. Participants were instructed to read at a natural pace ensuring comprehension and were not alerted to the presence of grammatical errors in the stimuli, but they were warned that sentences read out of context might seem pragmatically odd. The order of sentence presentation within each list was randomized for each participant. Four practice items were presented before the start of the experiment, one of which was ungrammatical and three of which were followed by a question.

### 2.4. Analysis

All data were analyzed in the R statistical software platform (R Core Team, 2015). Subjects were excluded if they answered <50% of the comprehension questions correctly. Only reaction time data from sentences in which the comprehension question was answered correctly were included for analysis. Previous work attentive to the contribution of different portions of the reaction time distribution to agreement attraction configurations has shown that the canonical comprehension attraction effects are contained disproportionately in the right tail of reading times in regions where effects exist (see Staub, 2009, 2010; Lago et al., 2015; Tucker et al., 2015; Almeida and Tucker, 2017; Villata et al., 2018). Therefore, we deliberately chose a conservative method of by-region outlier treatment: Winsorization at 1% of the by-region mean (see Ratcliff, 1993 for discussion). No other exclusion criteria were used.

### 2.5. Results

#### 2.5.1. Comprehension Question Accuracy

No participants met the criterion for exclusion due to low comprehension question accuracy for this experiment. Overall comprehension question accuracy across all subjects was 88.5% for all items, with an accuracy of 90.2% for fillers and 83.4% for experimental items.

#### 2.5.2. Self-Paced Reading

**Figure 1:**
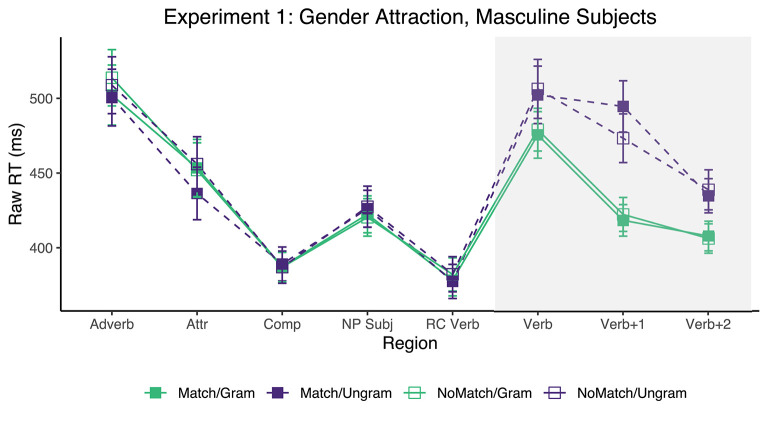
Mean raw reading times from Experiment 1 for all conditions and regions. Error bars represent the standard error of the condition mean across participant averages.

Only the sentences for which the comprehension question was answered correctly were included for analysis. This resulted in the exclusion of 12.80% of the raw collected data (across all conditions, regions, and participants). Mean reading times for each region and condition in Experiment 1 appear in Figure 1. The grammaticality and attraction effects were calculated as described in the introduction, and the results are presented in Table 2. There were substantial grammaticality effects in the *Verb* and two immediately subsequent regions (54, 127, and 59 ms, respectively). However, evidence for an attraction effect was only observed for ungrammatical sentences, and in the *Verb*+*1* region (21 ms). The 95% CI of the latter effect, however, did not exclude zero (95%CI [−1, 43] ms).

**Table 2:** Results of experiment 1.

**N = 104**	**Verb**		**Verb+1**		**Verb+2**	
Attraction ungrammatical	−4	(−29, 20)	*21*	*(−1, 43)*	−4	(−21, 10)
Attraction grammatical	−4	(−22, 14)	−4	(−16, 8)	2	(−8, 11)
Grammaticality	**54**	**(20, 93)**	**127**	**(96, 162)**	**59**	**(43, 78)**

*Mean RT for each effect of interest. 95% Confidence Intervals computed by BCa bootstrap (2,000 replications) in parenthesis. Effects in which the CI excludes zero are marked in bold. Effects in which the CI includes zero up to ±1 ms are marked in italic*.

### 2.6. Discussion

The results of Experiment 1 provide some evidence that gender, like number, can be confusable in comprehension. The longer reading times to regions including and following the main clause verb suggest that readers notice verb ungrammaticalities on the whole, spending longer time attempting to resolve the conflicting agreement information. However, relative to the baseline match condition, sentences with a mismatching attractor do not show such a marked slowdown. This facilitative interaction is the hallmark of agreement attraction effects in comprehension (see Pearlmutter et al., 1999; Wagers et al., 2009; Lago et al., 2015; Dillon et al., 2013; Tucker et al., 2015 and references therein). Moreover, these effects with gender are not seen in equal measure with grammatical verbs, providing evidence that gender attraction effects, much like number, show a grammaticality asymmetry (Wagers et al., 2009; Jäger et al., 2017).

However, this is only one-half of the attraction effect profile seen for number in languages, such as, e.g., English. The other component to this effect is an asymmetry owing to *markedness*—attraction effects on reaction times or in productions are often found in languages when the erroneous verbal morphology is the marked version more than when it is in the unmarked version (Eberhard, 1997). Since this is an important dimension upon which to assess the similarity of gender and number attraction, Experiments 2A and 2B, involving the manipulation of subject gender, were designed to address this question.

## 3. Experiments 2A and 2B: Gender Attraction and Morphological Markedness

Our goal in the second experiment was to assess whether the evidence of attraction effects for Arabic gender we obtained in experiment 1 is replicable, and if so, whether gender attraction effects would pattern along markedness lines the way agreement cues have been observed to in other languages. At least three papers (Badecker and Lewis, 2007; Badecker and Kuminiak, 2007; Slioussar and Malko, 2016) have shown that gender attraction can in principle follow language-internal markedness hierarchies with attraction effects sensitive to whether the verb appears in the marked or unmarked version. These findings are at odds, however, with findings from Hebrew, where markedness effects do not appear to obtain in production (Dank and Deutsch, 2010). Moreover, only one study (Slioussar and Malko, 2016) has assessed this phenomenon in comprehension, reporting one experiment on the three-way gender system of modern Russian.

In MSA—a language with a two-valued system including masculine and feminine nouns—the marked grammatical gender is arguably feminine given that on many nouns, feminine gender is overtly marked with a suffix. Furthermore, conjunctions containing both masculine and feminine nouns invariably resolve to the masculine plural (Ryding, 2005). We therefore expect to find that gender attraction effect profiles would appear more often in reading times when the true subject is masculine and the attractor feminine, rather than the other way around, if markedness effects obtain as in English number, where ungrammatical plural verbs are more acceptable with plural attractors than ungrammatical singular verbs with singular attractors.

### 3.1. Participants

Participants in experiment 2A were 128 native speakers of Arabic from the UAEU student community with no history of language disorder and self-assessed proficiency in MSA (128 females; mean age 20.4 years). Participants in the replication experiment 2B were 202 native speakers of Arabic from the UAEU student community with no history of language disorder and self-assessed proficiency in MSA (202 females; mean age 20.9 years). All other participant recruitment and consent procedures were identical to Experiment 1. No participants in any experiment reported in this study took part in more than one of the experiments.

### 3.2. Materials

The 48 item sets from Experiment 1 were altered to allow the main clause subject NP to also appear with the feminine suffix *-a*/ــة. Where pragmatics required, the continuations were altered to allow for sensible interpretations across different genders of subject NPs. Items which were identical to the items used in Experiment 1 save for these specific changes.

Using each of the 48 sentences as a standard, seven additional variants were constructed by systematically varying the grammatical gender of both the main clause subject and relative clause object NP as well as the main clause verb (the target verb). All feminine NPs were created by attaching the feminine suffix *-a*/ــة to the NP used in the equivalent masculine conditions. The result is eight conditions per experimental sentence in a 2 × 2 × 2 factorial design crossing Subject Gender, Grammaticality, and Match.

It should also be noted that complementizers in MSA agree with the NP they modify in both grammatical number and grammatical gender (Ryding, 2005, p. 322), meaning that conditions with a feminine subject also contain a feminine singular definite complementizer (ʔ*allatii*/التي), in contrast to the masculine singular definite complementizer (ʔ*allaðii*/الذي) found in masculine subject conditions. Additionally, whenever the subject NP was feminine, the relative clause verb also appeared in the feminine, so that the only possible agreement attraction effects occur on the main clause/target verb. This procedure was followed to construct items for all subsequent experiments, as well. A complete item set for one experimental sentence appears in Table 3.

**Table 3:** A complete item set for one stimulus in Experiment 2.

**Condition**	**NP1**	**Comp**	**RCV**	**NP2**	**Adv**	**V**	**Continuation**
	**R1**	**R2**	**R3**	**R4**	**R5**	**R6**	**R7–R_**n**_**
MASC/MATCH/GRAM	المهندس	الذي	استقبل	العالِم	بالصدفةِ	يعمل	.على ابتكار جديد
	The engineer (MASC)	who	met	the scientist (MASC)	by chance	is working (MASC)	on a new invention.
MASC/MATCH/UNGRAM	المهندس	الذي	استقبل	العالِم	بالصدفةِ	تعمل	.على ابتكار جديد
	The engineer (MASC)	who	met	the scientist (MASC)	by chance	is working (FEM)	on a new invention.
MASC/NOMATCH/GRAM	المهندس	الذي	استقبل	العالِمة	بالصدفةِ	يعمل	.على ابتكار جديد
	The engineer (MASC)	who	met	the scientist (FEM)	by chance	is working (MASC)	on a new invention.
MASC/NOMATCH/UNGRAM	المهندس	الذي	استقبل	العالِمة	بالصدفةِ	تعمل	.على ابتكار جديد
	The engineer (MASC)	who	met	the scientist (FEM)	by chance	is working (FEM)	on a new invention.
FEM/NOMATCH/GRAM	المهندسة	التي	استقبلت	العالِم	بالصدفةِ	تعمل	.على ابتكار جديد
	The engineer (FEM)	who	met	the scientist (MASC)	by chance	is working (FEM)	on a new invention.
FEM/NOMATCH/UNGRAM	المهندسة	التي	استقبلت	العالِم	بالصدفةِ	يعمل	.على ابتكار جديد
	The engineer (FEM)	who	met	the scientist (MASC)	by chance	is working (MASC)	on a new invention.
FEM/MATCH/GRAM	المهندسة	التي	استقبلت	العالِمة	بالصدفةِ	تعمل	.على ابتكار جديد
	The engineer (FEM)	who	met	the scientist (FEM)	by chance	is working (FEM)	on a new invention.
FEM/MATCH/UNGRAM	المهندسة	التي	استقبلت	العالِمة	بالصدفةِ	يعمل	.على ابتكار جديد
	The engineer (FEM)	who	met	the scientist (FEM)	by chance	is working (MASC)	on a new invention.

The 48 sets of eight sentences were distributed across eight lists in a Latin Square design after being combined with 144 grammatical fillers of a similar length for a 3:1 filler-to-item ratio. None of the fillers used in Experiment 1 were used for this experiment, and all other filler constraints were identical to Experiment 1. Only the experimental sentences contained ungrammaticalities (12.5% of the sentences in each list).

### 3.3. Procedure

The procedure for Experiments 2A and 2B were identical to that employed for Experiment 1, save for the difference that participants in 2A were asked to participate in a second, unrelated experiment upon completion of the self-paced reading experiment reported here.

### 3.4. Analysis

Comprehension question accuracy data in Experiments 2A and 2B were analyzed identically as in Experiment 1. For the self-paced reading data, all of the analysis was the same as Experiment 1 save for the inclusion of the additional experimental manipulation of Subject Gender. Thus, the effects of interest are still computed as described in the introduction, except that they are calculated along the levels of subject gender.

### 3.5. Results

#### 3.5.1. Comprehension Question Accuracy

In experiment 2A, three participants failed to meet the comprehension question accuracy criterion and were excluded from this and all further analysis. Overall comprehension question accuracy for this experiment was 86.7%, with an accuracy of 87.7% for fillers and 83.7% for experimental items. In experiment 2B, only one participant failed to meet the comprehension question accuracy criterion and was excluded from this and all further analysis. Overall comprehension question accuracy for the replication was 86.2%, with an accuracy of 85.0% for the experimental items and 87.9% for fillers.

#### 3.5.2. Self-Paced Reading

**Figure 2:**
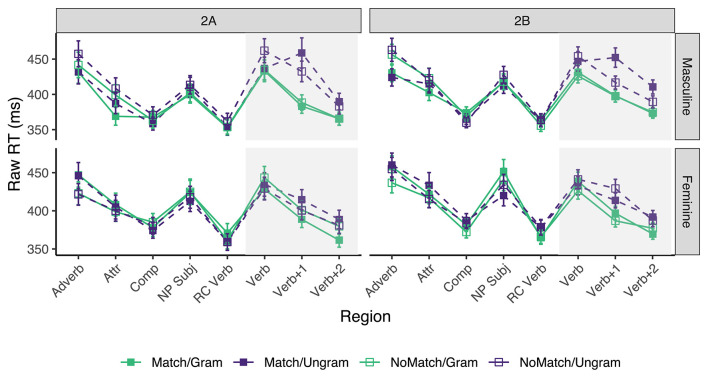
Mean raw reading times from Experiment 2 for all conditions and regions by Subject Gender. Error bars represent the standard error of the condition mean across participant averages.

Only the sentences for which the comprehension question was answered correctly were included. This resulted in the exclusion of 14.56% of the raw collected data (across all conditions, regions, and participants) in Experiment 2A, and 16.7% of the data in Experiment 2B. Mean reading times for each region and condition in Experiments 2A and 2B appear in Figure 2. The grammaticality and attraction effects were calculated as described in the introduction, and the results are presented in Table 4.

**Table 4:** Results of experiment 2.

	**Subject**	**Verb**		**Verb+1**		**Verb+2**	
**2A: N = 125**							
Attraction ungrammatical	Masc	−25	(−48, −2)	26	(−4, 63)	7	(−6, 21)
	Fem	5	(−17, 29)	14	(−4, 32)	8	(−7, 23)
Attraction grammatical	Masc	−1	(−26, 22)	−5	(−20, 8)	0	(−13, 12)
	Fem	−15	(−33, 2)	*−12*	*(−30, 1)*	**−18**	**(−32**, **−6)**
Grammaticality	Masc	30	(−7, 71)	**119**	**(82, 163)**	**42**	**(20, 64)**
	Fem	−8	(−42, 25)	**25**	**(2, 49)**	**28**	**(9, 49)**
**2B: N = 201**							
Attraction ungrammatical	Masc	−7	(−26, 12)	**35**	**(17, 57)**	**21**	**(6, 38)**
	Fem	−8	(−26, 8)	*−16*	*(−40, 1)*	4	(−7, 14)
Attraction grammatical	Masc	5	(−10, 23)	1	(−13, 14)	−2	(−12, 9)
	Fem	12	(−3, 29)	10	(−2, 24)	−7	(−18, 3)
Grammaticality	Masc	**43**	**(14, 74)**	**73**	**(51, 99)**	**52**	**(34, 72)**
	Fem	9	(−15, 36)	**59**	**(33, 89)**	**33**	**(16, 49)**

*Mean RT for each effect of interest. 95% Confidence Intervals computed by BCa bootstrap (2,000 replications) in parenthesis. Effects in which the CI excludes zero are marked in bold. Effects in which the CI includes zero up to ±1 ms are marked in italic*.

In experiment 2A, a grammaticality effect for sentences with masculine subjects appeared in all three critical regions (30, 119, and 42 ms, respectively) although only in the latter two did the CI exclude 0. Smaller grammaticality effects were also observed for sentences with feminine subjects, but only in the *Verb*+*1* and *Verb*+*2* regions (25 and 28 ms, respectively). As for the attraction effect, we find a numerical trend only in grammatical sentences in the *Verb*+*1*, and to a lesser extent, *Verb*+*2* regions, even though in none of these regions the 95% CI excludes zero. The effect size for the attraction effect is larger for ungrammatical sentences with masc subjects (26 ms) than for fem subjects (14 ms) in the *Verb*+*1* region, and equivalent in the *Verb+2* region. In addition, for the grammatical sentences, we observe a “reverse” attraction effect in almost all critical regions, but only in the *Verb*+*2* region of sentences with fem subjects did the CI exclude 0 (−18 ms).

In experiment 2B, we find grammaticality effects starting in the *Verb* and continuing into the two subsequent regions for sentences with masculine subjects (43, 73, and 52 ms, respectively). The grammaticality effect in sentences with feminine subjects started in the *Verb*+*1* region and continues into the *Verb+2* region (59 and 33 ms, respectively). The attraction effect in experiment 2B was only reliably observed in ungrammatical sentences, and within this group, only in sentences with masculine subjects. It starts in the *Verb*+*1* region (35 ms) and continues into the subsequent region (21 ms). Contrary to the results of experiment 2A, ungrammatical sentences with fem subjects had a “reverse” attraction effect in the *Verb*+*1* region (−16 ms), and the “reverse” attraction effect observed in grammatical sentences flipped sign in the first two critical regions.

### 3.6. Discussion

Experiments 2A and 2B offer some indication of a markedness asymmetry in verbal gender agreement attraction: in experiment 2A, feminine-subject ungrammatical sentences showed a modest attraction effect (14 ms, though the 95% CI did not exclude zero) in the *Verb*+*1* region. However, this effect was not replicated in experiment 2B, where it in fact became a “reverse” attraction effect of −16 ms. This is unlike the results observed in masculine-subject sentences across the three experiments reported thus far, which showed remarkable consistency in effect sizes in that same post-verbal region (21, 26, 35 ms). As for the grammaticality asymmetry, the combined results of experiments 1, 2A, and 2B show that the attraction effect seems to be more reliably elicited in ungrammatical sentences, mirroring the findings for what has been observed for number in languages like English (Wagers et al., 2009).

## 4. Experiment 3: Number Attraction and Morphological Realization

In order to examine the similarities and differences between gender and number attraction in MSA, one must examine whether the markedness asymmetry is present in Arabic number attraction—an effect left untested in the only comprehension study in language, reported by Tucker et al. (2015). Due to grammatical factors, testing number independent of gender in Arabic requires making a choice about which genders to include while independently manipulating number values. Since gender is orthogonal to number in MSA number agreement paradigms, the simplest option would be to simply counterbalance masculine and feminine verbs across experimental items. However, Tucker et al. (2015) presents findings concerning the potential interplay of nominal gender and morphophonological effects on plural formation which make this counterbalancing possibly undesirable. Since any experiment which *a priori* restricted itself to one of two available genders in a language would need to be justified, we first examine the findings from Tucker et al. (2015) in some detail with an experiment designed to replicate and extend those findings.

Arabic allows for two different strategies of plural formation: sound/suffixing plurals and broken/ablauting plurals. The former take their plurals with a regular, shape-invariant suffix (in that study, *-aat*/ات-), whereas the latter mark plurality by a change in the vowel and syllabic structure of the singular noun. The vast majority of words in Arabic can be decomposed into a CV-template and root consisting of 2–4 consonants, in the root $\sqrt{\text{drs}}$ common to words, such as *darasa*/درس, “he studied;” *darrasa*/درّس, “he taught;” and *madrasa*/مدرسة—“school” (Wehr, 1976, p. 321).

Typologically, Arabic is unique in the high number of broken/ablauting plurals relative to other languages which utilize alteration of the CV-template—indeed, they are arguably more frequent than suffixing/sound plurals insofar as many of the high-frequency nouns in the language take broken plurals (but see discussion in Boudelaa and Gaskell, 2002). Here, examining just English would lead to a different conclusion, such as that reached by Bock and Eberhard (1993), who demonstrate that attractors with irregular plurals in English do not condition different attraction rates in production than those with regular plurals.

As Ryding (2005) and Tucker et al. (2015) note, masculine animate nouns tend to take broken plurals and feminine animate nouns tend to take sound plurals. In Tucker et al. (2015), the data indicated that ablaut plural attractors (all masculine in the study) cause smaller intrusion effect sizes at ungrammatical verbs than sound plural attractors (all feminine in the study) do. Given that all the subjects in this experiment were singular, Tucker et al. reason that this might be due to the salience of morphological plural marking on the attractor insofar as sound plurals contain a morphological or orthographic unit (the suffix) which is clearly associated with plurality, whereas comprehension of a broken plural *qua* plural requires decomposition of a word into its root and CV-template.

However, one issue that study does not address is whether there might be differentiations to be made inside the class of broken plurals such that the distinction in attraction effect sizes is not due to broken plurals *per se*, but instead the ambiguity of the morphological marking: sound plural suffixes *unambiguously* mark plural number, whereas template alterations mark many morphological distinctions. Whether morphophonological properties of the attractor plays a role in modulating attraction rates is currently an open question at present: Vigliocco et al. (1995) and Slioussar and Malko (2016) find that they do not, Badecker and Kuminiak (2007), Dank and Deutsch (2010) and to some extent Hartsuiker et al. (2003) find that they do.

In order to test this hypothesis, we manipulate the morphological ambiguity of the CV-templates used to mark plural on attractors. For example, the CV-template associated with the plural noun لصوص/*lus*^ʕ^*uus*^ʕ^, “thieves”—C_1_uC_2_uuC_3_—is also found in singular nouns, such as the deverbal nominalization دخول/*duxuul*, “entering (n.)” and is therefore morphologically ambiguous with respect to number marking. This can be contrasted with a different template—such as C_1_uC_2_aC_3_aaʔ as in the noun علماء/ʕ*ulamaa*ʕ, “scientists”—which is found only with plural nouns and can be considered morphologically unambiguous with respect to number.

### 4.1. Participants

Participants were 110 native speakers of Arabic from the UAEU community (110 females; mean age 21.1 years).

### 4.2. Materials and Predictions

Forty-eight sentences were constructed exactly as in the previous two experiments and in Tucker et al. (2015). This is twice the number of items with masculine pre-critical NPs compared to the subgroup in Tucker et al. (2015), where only 24 such items appeared. In this experiment, however, both *NP1* and *NP2* were specified as masculine grammatically and took their plural form in a broken pattern and not with a suffix. Additionally, broken plurals were classified into two categories—ambiguous and unambiguous plurals. Plural ambiguity was assigned based on the prosodic/CV-template pattern that the plural contained. Templates were considered ambiguous if the second author and a collection of other native speaker consultants could easily think of singular nouns which appeared in that same CV-pattern and unambiguous otherwise. A complete list of the templates and classifications used in the construction of the stimuli for this experiment appear in the Supplementary Material. In order to keep the duration of the experiment manageable, the ambiguity of NP2 was manipulated across the 48 sentences. The result was 24 items with NP2s that took ambiguous plurals and 24 items with NP2s that took unambiguous plurals. All other constraints on the creation of stimuli in Experiments 1 and 2 were followed, where applicable to number instead of grammatical gender.

The 48 sentences were then individually converted into four conditions by systematically varying the grammatical number (singular, plural) of both NP2 and the target verb. The resulting collection of four conditions for each of the 48 sentences comprised a 2 × 2 × 2 factorial design crossing Match (yes, no) and Grammaticality (grammatical, ungrammatical) and Plural Template Ambiguity (ambiguous, unambiguous). In this study, all the NoMatch conditions contained a singular NP1 and a plural NP2, and ungrammatical verbs were always plural. These 48 sets of four sentences were distributed across four lists in a Latin Square design and combined with 144 grammatical fillers for a 3:1 filler:item ratio where 12.5% of the items were ungrammatical.

If the diminished attraction effect reported by Tucker et al. (2015) in masculine items with ablaut plurals is due to a inherent difference between pluralization processes (ablaut vs. suffixation), then the prediction would be that neither ambiguous nor unambiguous ablaut plurals would exhibit attraction effects. If, however, it is the ambiguity of the plural marking of ablaut forms that is responsible for the diminishing of attraction effects reported by Tucker et al. (2015) in masculine broken plurals, then we would expect to observe number attraction effects for unambiguous broken plurals, but not for ambiguous ones.

### 4.3. Procedure

The procedure for Experiment 3 was exactly the same as the procedure for Experiments 2A and 2B.

### 4.4. Analysis

Comprehension question accuracy data for Experiment 3 was analyzed identically to the analysis of experiments 1, 2A, and 2B. For the self-paced reading data, all of the analysis was the same as Experiment 1 save for the addition of the additional experimental manipulation of plural template ambiguity of the attractor NP. Thus, the effects of interest were computed as described in the introduction, except that they were calculated along the levels of plural template ambiguity.

### 4.5. Results

#### 4.5.1. Comprehension Question Accuracy

None of the participants in this experiment met the criteria for exclusion based on global comprehension question accuracy, and so all were included in the subsequent analyses. Overall comprehension question accuracy for this experiment was 88.8% with accuracy rates of 86.8% for fillers and 89.5% for experimental items.

#### 4.5.2. Self-Paced Reading

**Figure 3:**
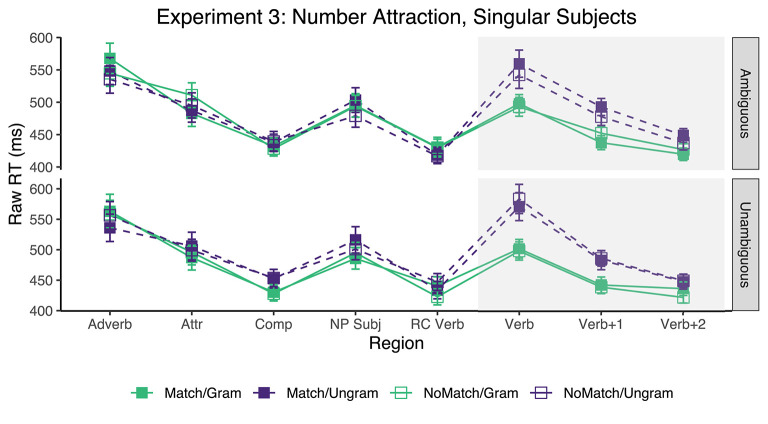
Mean raw reading times from Experiment 3 for all conditions and regions by attractor Ambiguity. Error bars represent the standard error of the condition mean across participant averages.

Only sentences for which the comprehension question was answered accurately were included, resulting in the exclusion of ~13.01% of the raw collected data (across all conditions, participants, and items). Mean reading times across participant averages for each region are shown in Figure 3. Table 5 shows the results for critical regions of interest.

**Table 5:** Results of experiment 3.

**N = 110**	**Verb**		**Verb+1**		**Verb+2**	
**AMBIGUOUS**						
Attraction ungrammatical	17	(−15, 50)	15	(−9, 38)	12	(−6, 32)
Attraction grammatical	5	(−16, 26)	−15	(−35, 4)	−7	(−23, 9)
Grammaticality	**110**	**(67, 159)**	**80**	**(46, 116)**	**39**	**(13, 67)**
**UNAMBIGUOUS**						
Attraction ungrammatical	−13	(−51, 22)	−3	(−29, 31)	−1	(−19, 17)
Attraction grammatical	4	(−24, 28)	4	(−16, 22)	14	(−2, 32)
Grammaticality	**154**	**(99, 216)**	**88**	**(50, 124)**	**39**	**(13, 67)**

*Mean RT for each effect of interest. 95% Confidence Intervals computed by BCa bootstrap (2,000 replications) in parenthesis. Effects in which the CI excludes zero are marked in bold. Effects in which the CI includes zero up to ±1 ms are marked in italic*.

The only reliable results observed here were the grammaticality effects, which were found in the *Verb* and its two subsequent regions for sentences containing attractors carrying both ambiguous and unambiguous plural templates.

### 4.6. Discussion

Experiment 3 provides a replication of one-half of the experiment reported in Tucker et al. (2015): it contained items with masculine NPs and attractors that take broken plurals. Like Tucker et al. (2015), we fail to find any reliable evidence of attraction effects in reading times for such items. The only effects that were numerically compatible with number attraction were the ones from sentences that, contrary to our hypothesis, had *ambiguous* attractors, although in none of them did the 95% CIs come close to excluding zero in the three critical regions. Because grammaticality differences are being noticed by participants regardless of the attractor type, it is clear that participants are attending to the agreement morphology; they just do not seem to be subject to sufficiently strong attraction effects when the attractor is an ablaut/broken plural.

Therefore, in this experiment we find no evidence that it was the morphological ambiguity of the broken plural template of the attractor that was responsible for the observed difference in attraction rates between sound and broken plural attractors in Tucker et al. (2015). This result leaves open the possibility that it is type of pluralization (ablaut vs. suffixation) which was responsible for that outcome, and is also converging evidence with results reported by, for instance, Vigliocco et al. (1995) and Slioussar and Malko (2016), that morphological ambiguity of the attractor not relating to case morphology plays little or no role in modulating attraction rates.

## 5. Experiments 4A and 4B: Number Attraction and Morphological Markedness

While Experiment 3 seems to confirm the finding that MSA number agreement attraction is either not present or drastically reduced when the pre-critical region contains masculine NPs and/or broken plural attractors (two potential causal variables that are inextrincably highly correlated in the language), there are still two open questions about the nature of number agreement attraction in MSA given the results from Tucker et al. (2015) and the first four experiments reported here. First, while it has been claimed above that gender attraction effects mirror agreement attraction effects in directionality and potentially markedness as well, this latter property has not been evaluated for Arabic number agreement. The predictions are clear: given that English number attraction only gives rise to attraction RT profiles when the unmarked singular (*is*) is replaced by the marked plural (*are*), one could expect that attraction proceeds in the same way in MSA. In addition, given that English and Arabic belong to distinct language families where different notions of markedness are could be at play, it is important to examine whether plural-to-singular attractions give rise to attraction RT profiles in MSA, as well. To these ends, we designed an experiment exactly like Experiments 2A and 2B, but which utilized only the feminine/sound plural attractor subgroup of items from Tucker et al. (2015). This choice is motivated by the combined results of Tucker et al. (2015) and our experiment 3, which raise the question of whether masculine/broken plural attractors induce attraction rates to the same extent as feminine/sound plural attractors. The result is an experiment designed to replicate the presence of attraction for number cues at the verb while simultaneously testing for the presence or absence of a markedness asymmetry in MSA number agreement attraction effects.

### 5.1. Participants

Participants in experiment 4A were 112 native speakers of Arabic from the UAEU community (112 females; mean age 20.6 years). Participants in experiment 4B were 218 native speakers of Arabic from the UAEU community (218 females; mean age 20.6 years).

### 5.2. Materials and Predictions

54 sentences conforming to the constraints in the previous three experiments were constructed along the lines described in the introduction. However, in this experiment both *NP1* and *NP2* were constrained to be grammatically feminine nouns bearing the feminine suffix *-a*/ــة. Given that these nouns had singulars ending in *-a*/ــة, their plurals were all suffixal, ending in *-aat*/ات-.

The sentences were then individually converted into eight conditions by systematically varying the grammatical number (singular or plural) of the word in the *NP1* position, as well as the appropriate Match and Grammaticality properties of the items. The result was a collection of eight variants organized in a 2 × 2 × 2 factorial design crossing Subject Number (singular, plural), Match, and Grammaticality. A complete item set for one of the experimental sentences appears in Table 6 and a complete list of experimental sentences appears in Supplementary Material.

**Table 6:** A complete item set for one stimulus in Experiment 4.

**Condition**	**NP1**	**Comp**	**RCV**	**NP2**	**Adv**	**V**	**Continuation**
	**R1**	**R2**	**R3**	**R4**	**R5**	**R6**	**R7–R_**n**_**
SG/MATCH/GRAM	المدربة	التي	اهتمت	باللاعبة	جداً	اشتغلت	.في الأكاديمية الوطنية للمبارزة
	The coach (SG)	who	was interested	in.the player (SG)	very	worked (SG)	at the National Fencing Academy.
SG/MATCH/UNGRAM	المدربة	التي	اهتمت	باللاعبة	جداً	اشتغلن	.في الأكاديمية الوطنية للمبارزة
	The coach (SG)	who	was interested	in.the player (SG)	very	worked (PL)	at the National Fencing Academy.
SG/NOMATCH/GRAM	المدربة	التي	اهتمت	باللاعبات	جداً	اشتغلت	.في الأكاديمية الوطنية للمبارزة
	The coach (SG)	who	was interested	in.the players (PL)	very	worked (SG)	at the National Fencing Academy.
SG/NOMATCH/UNGRAM	المدربة	التي	اهتمت	باللاعبات	جداً	اشتغلن	.في الأكاديمية الوطنية للمبارزة
	The coach (SG)	who	was interested	in.the players (PL)	very	worked (PL)	at the National Fencing Academy.
PL/NOMATCH/GRAM	المدربات	اللواتي	اهتمن	باللاعبة	جداً	اشتغلن	.في الأكاديمية الوطنية للمبارزة
	The coach (PL)	who	were interested	in.the player (SG)	very	worked (PL)	at the National Fencing Academy.
PL/NOMATCH/UNGRAM	المدربات	اللواتي	اهتمن	باللاعبة	جداً	اشتغلت	.في الأكاديمية الوطنية للمبارزة
	The coach (PL)	who	were interested	in.the player (SG)	very	worked (SG)	at the National Fencing Academy.
PL/MATCH/GRAM	المدربات	اللواتي	اهتمن	باللاعبات	جداً	اشتغلن	.في الأكاديمية الوطنية للمبارزة
	The coach (PL)	who	were interested	in.the players (PL)	very	worked (PL)	at the National Fencing Academy.
PL/MATCH/UNGRAM	المدربات	اللواتي	اهتمن	باللاعبات	جداً	اشتغلت	.في الأكاديمية الوطنية للمبارزة
	The coach (PL)	who	were interested	in.the players (PL)	very	worked (SG)	at the National Fencing Academy.

*Note that NP1, NP2, RCV, and V are all morphologically feminine*.

These 54 sets of eight sentences were distributed across eight lists in a Latin Square design and combined with 144 fillers for a filler-to-item ratio of 2.67:1. The fillers were randomly selected from the collection of fillers used in Experiments 1–3 for this purpose. All the fillers were grammatical with a total of 13.6% of the sentences ungrammatical in any given list.

If the results from the subset of items in Tucker et al. (2015) bearing feminine sound plural attractors replicate, then one expects to find a grammaticality effect beginning at the main clause/target verb along with the number attraction effect. These effects may spill over into the post-verbal regions but, given the effects in the previous study by Tucker et al., one expects to find that the number attraction effect begins and is largest at the critical verb region itself.

### 5.3. Procedure

The procedure followed for Experiment 4 was exactly the same as the procedure for Experiments 1, 2A, 2B, and 3.

### 5.4. Analysis

Comprehension question accuracy for Experiments 4A and 4B were analyzed identically to the comprehension question accuracy analysis in Experiments 1–3. For the self-paced reading data, raw reading times were analyzed exactly as in Experiments 2A and 2B, save for the substitution of Subject Gender for Subject Number.

### 5.5. Results

#### 5.5.1. Comprehension Question Accuracy

In Experiment 4A, one subject met the criteria for exclusion due to low accuracy based upon global comprehension question scores; she was excluded from the subsequent analyses. Overall comprehension question accuracy for this experiment was 89.6% with accuracy rates of 89.4% for fillers and 89.7% for experimental items. In Experiment 4B, two subjects met the criteria for exclusion due to low accuracy based upon global comprehension question scores; they were also excluded from the subsequent analyses. Overall comprehension question accuracy for the replication was 87.6% with accuracy rates of 87.6% for both experimental and filler items, respectively.

#### 5.5.2. Self-Paced Reading

**Figure 4:**
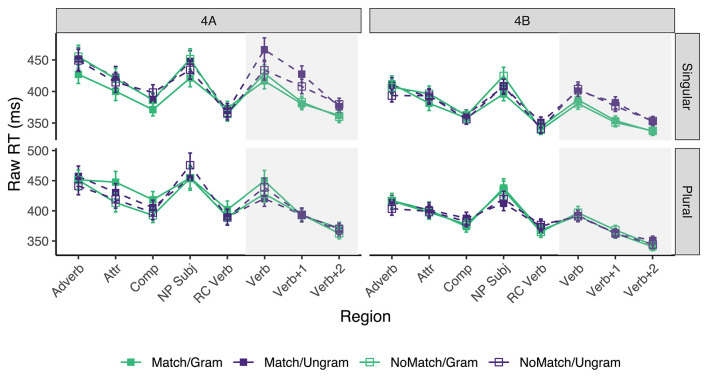
Mean raw reading times from Experiment 4 for all conditions and regions by subject number. Error bars represent the standard error of the condition mean across participant averages.

Only sentences for which the comprehension questions were answered correctly were included, resulting in the exclusion of ~10.69% of the raw data acquired from the experimental sentences (across all conditions, participants, and items) in experiment 4A, and 12.7% in experiment 4B. Mean reading times across participant averages for all conditions by subject number appear in Figure 4. Table 7 shows the results for critical regions of interest.

**Table 7:** Results of experiment 4.

	**Subject**	**Verb**		**Verb+1**		**Verb+2**	
**4A: N = 111**							
Attraction ungrammatical	Singular	**32**	**(10, 56)**	**19**	**(2, 40)**	−4	(−17, 9)
	Plural	*−19*	*(−39, 1)*	0	(−13, 13)	−3	(−15, 8)
Attraction grammatical	Singular	−12	(−28, 7)	−3	(−14, 10)	2	(−9, 14)
	Plural	*21*	*(−1, 46)*	−1	(−21, 15)	9	(−2, 21)
Grammaticality	Singular	**55**	**(21, 100)**	**72**	**(50, 96)**	**33**	**(15, 55)**
	Plural	−18	(−50, 13)	1	(−22, 21)	2	(−16, 18)
**4B: N = 216**							
Attraction ungrammatical	Singular	−3	(−16, 10)	6	(−6, 19)	−1	(−10, 7)
	Plural	1	(−10, 12)	0	(−8, 10)	*7*	*(−1, 16)*
Attraction grammatical	Singular	6	(−6, 18)	3	(−5, 12)	−1	(−9, 6)
	Plural	−5	(−18, 7)	−6	(−15, 4)	−3	(−11, 5)
Grammaticality	Singular	**37**	**(17, 58)**	**54**	**(39, 72)**	**31**	**(18, 44)**
	Plural	−6	(−26, 14)	−5	(−19, 9)	*11*	*(0, 24)*

*Mean RT for each effect of interest. 95% Confidence Intervals computed by BCa bootstrap (2,000 replications) in parenthesis. Effects in which the CI excludes zero are marked in bold. Effects in which the CI includes zero up to ±1 ms are marked in italic*.

In experiments 4A and 4B alike, reliable grammaticality effects were only observed in singular subject sentences, and they were found in the three critical regions. As for the presence of number attraction effects, in experiment 4A we find strong effects in the *Verb* (32 ms) and *Verb*+*1* (19 ms) regions, but only for ungrammatical sentences with singular subjects; no reliable effects were observed when the subject was plural or the sentence was grammatical. In experiment 4B, there was no reliable evidence of number attraction effects in neither grammaticality condition.

### 5.6. Discussion

The results of Experiment 4A largely replicate the results found by Tucker et al. (2015) for the feminine suffixing plural subgroup of items. Specifically, participants are able to recognize grammaticality manipulations early—upon being presented with the ungrammatical verb. Also like in all experiments in this study, the grammaticality effect is also found in post-verbal regions. The number attraction effect which appears at the verb for singular subject sentences is a direct analog of the attraction effect in English and a replication of the previous results reported by Tucker et al. (2015). Moreover, this effect is largest at the verb region and continues into the immediately post-verbal spillover region. Moreover, the results for experiment 4A also show other properties normally associated with number attraction in other languages: the grammaticality asymmetry and the markedness asymmetry are both present.

For all these reasons, it is perplexing that the results of experiment 4B fail to replicate the attraction effect observed in Tucker et al. (2015) and in experiment 4A, even though a grammaticality effect is observed at the verb and all post-verbal critical regions. This discrepancy is larger in the *Verb* region, where the attraction effect in ungrammatical singular subject sentences flips signs between experiments. The discrepancy in the *Verb*+*1* region is easier to reconcile, as the effects trend numerically in the same direction and are included within each other's CIs: the 19 ms effect in experiment 4A is included in experiment 4B's CI [−6, 19], and the 6 ms effect in experiment 4B is included in the experiment 4A's CI [2, 40]. We return to this issue in discussion of the meta-analysis, presented below. However, given the failure to reliably replicate number attraction effects in experiment 4B, it is important to see if number and gender attraction effect differences can occur within the same experiment, for the same population of participants. This is the primary goal of experiments 5A and 5B.

## 6. Experiment 5A and 5B: Comparing Number and Gender Attraction

The results of experiments 1, 2A, and 2B thus far paint a consistent picture about the nature of gender attraction effects: They (i) exhibit a grammaticality asymmetry, (ii) exhibit a markedness asymmetry, and (iii) systematically occur after the *Verb* region, even though a grammaticality effect is often detectable at the *Verb* region itself.

The picture that emerges from Tucker et al. (2015) and experiments 3, 4A, and 4B about number attraction, on the other hand, is more mixed: when it occurs, it (i) exhibits a grammaticality asymmetry, (ii) exhibits a markedness asymmetry, (iii) systematically occurs at the *Verb* region (with potential spillover to the post-verbal region), and (iv) tends to occur only when the attractor features a suffix (instead of plural formation by ablaut).

These differences in timing (and perhaps reliability) observed between agreement attraction for number and gender have so far only been observed across different experiments, with different samples of participants. Therefore, it is important to see if the differences would hold in a fully within-participants design. That is the goal of experiment 5A. Given the importance of these findings, we again conduct a direct replication study (5B), with a different sample of participants.

### 6.1. Participants

Participants in experiment 5A were 200 native speakers of Arabic from the UAEU community (200 females; mean age 20.6 years). Participants in experiment 5B were another 100 native speakers of Arabic from the UAEU community (100 females; mean age 20.4 years).

### 6.2. Materials

The experimental items in experiments 5A and 5B were 54 sentences were manipulated into eight conditions by systematically varying the agreement features of *NP1, NP2*, and the *Verb* to recreate four conditions from Experiment 2A/2B and four conditions from Experiment 4A/4B—the conditions in which *NP1* bore the unmarked morphological value for both gender (masculine) and number (singular). The result was a collection of eight sentences organized in a 2 × 2 × 2 factorial design crossing: (1) Subject-Phi (Feature) (number, gender), (2) Match, and (3) Grammaticality. Because number attraction was the most robust with feminine attractors (*cf*. Experiments 3, 4A, and 4B), the number conditions feature feminine nouns in *NP1* and *NP2*. In the Gender conditions, *NP1* was always masculine and *NP2* varied in gender according to whether Match was *yes* or *no*. A complete set of sentences for one item appears in Table 8.

**Table 8:** A complete item set for one stimulus in Experiment 5.

**Condition**	**NP1**	**Comp**	**RCV**	**NP2**	**Adv**	**V**	**Continuation**
	**R1**	**R2**	**R3**	**R4**	**R5**	**R6**	**R7–R_**n**_**
NUM/MATCH/GRAM	المدربة	التي	اهتمت	باللاعبة	جداً	اشتغلت	.في الأكاديمية الوطنية للمبارزة
	The coach (FS)	who	was interested	in.the player (FS)	very	worked (FS)	at the National Fencing Academy.
NUM/MATCH/UNGRAM	المدربة	التي	اهتمت	باللاعبة	جداً	اشتغلن	.في الأكاديمية الوطنية للمبارزة
	The coach (FS)	who	was interested	in.the player (FS)	very	worked (FP)	at the National Fencing Academy.
NUM/NOMATCH/GRAM	المدربة	التي	اهتمت	باللاعبات	جداً	اشتغلت	.في الأكاديمية الوطنية للمبارزة
	The coach (FS)	who	was interested	in.the players (FP)	very	worked (FS)	at the National Fencing Academy.
NUM/NOMATCH/UNGRAM	المدربة	التي	اهتمت	باللاعبات	جداً	اشتغلن	.في الأكاديمية الوطنية للمبارزة
	The coach (FS)	who	was interested	in.the players (FP)	very	worked (FP)	at the National Fencing Academy.
GEN/MATCH/GRAM	المدرب	الذي	اهتم	باللاعبة	جداً	اشتغل	.في الأكاديمية الوطنية للمبارزة
	The coach(MS)	who	was interested	in.the player (MS)	very	worked (MS)	at the National Fencing Academy.
GEN/MATCH/UNGRAM	المدرب	الذي	اهتم	باللاعبة	جداً	اشتغلت	.في الأكاديمية الوطنية للمبارزة
	The coach (MS)	who	was interested	in.the player (MS)	very	worked (FS)	at the National Fencing Academy.
GEN/NOMATCH/GRAM	المدرب	الذي	اهتم	باللاعبات	جداً	اشتغل	.في الأكاديمية الوطنية للمبارزة
	The coach (MS)	who	was interested	in.the players (FS)	very	worked (MS)	at the National Fencing Academy.
GEN/NOMATCH/UNGRAM	المدرب	الذي	اهتم	باللاعبات	جداً	اشتغلت	.في الأكاديمية الوطنية للمبارزة
	The coach (MS)	who	was interested	in.the players (FS)	very	worked (FS)	at the National Fencing Academy.

*Note that NP1, NP2, RCV, and V are all morphologically feminine in the number manipulation and singular in the gender manipulation*.

These 54 sets of eight sentences were distributed across eight lists in a Latin Square design and combined with 144 fillers for a filler-to-item ratio of 2.67:1. The fillers were randomly selected from the collection of fillers used in Experiments 1–4B. All the fillers were grammatical with a total of 13.6% of the sentences ungrammatical in any given list.

### 6.3. Analysis

Comprehension question accuracy for Experiments 5A and 5B were analyzed identically to the comprehension question accuracy analysis in Experiments 1–4. For the self-paced reading data, raw reading times were analyzed exactly as in Experiments 4A and 4B, save for the substitution of Subject Number for Subject Phi.

### 6.4. Results

#### 6.4.1. Comprehension Question Accuracy

In Experiment 5A, two subjects met the exclusion criteria for low comprehension question accuracy scores; they were therefore excluded from any further analysis. Overall comprehension question accuracy for this experiment was 85.9%, with accuracy rates of 86.6% for fillers and 85.7% for experimental items. In Experiment 5B, four subjects met the exclusion criteria for low comprehension question accuracy scores; they were therefore excluded from this and any further analysis. Overall comprehension question accuracy for the replication was 86.5%, with accuracy rates of 86.6% for fillers and 86.4% for experimental items.

#### 6.4.2. Self-Paced Reading

**Figure 5:**
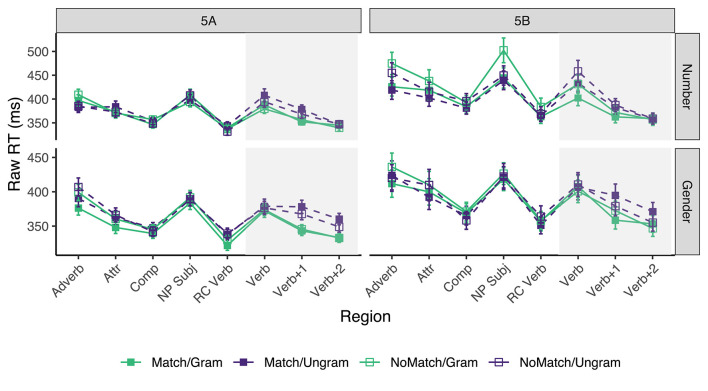
Mean raw reading times from Experiment 4 for all conditions and regions by subject number. Error bars represent the standard error of the condition mean across participant averages.

Only sentences for which the comprehension questions were answered correctly were included in the reading time analysis. This resulted in the exclusion of ~17.6% of the raw data acquired from the experimental sentences (across all conditions, participants, and items) in experiment 5A, and 17.9% in experiment 5B. Mean reading times across participant averages for all conditions by subject number appear in Figure 5. Table 9 shows the results for critical regions of interest.

**Table 9:** Results of experiment 5.

	**Subject**	**Verb**		**Verb+1**		**Verb+2**	
**5A: N = 198**							
Attraction ungrammatical	Gender	2	(−11, 15)	*10*	*(−1, 23)*	**11**	**(3, 21)**
	Number	14	(−2, 29)	10	(−3, 23)	0	(−11, 10)
Attraction grammatical	Gender	−2	(−11, 10)	−2	(−10, 6)	1	(−7, 9)
	Number	8	(−5, 21)	−4	(−14, 5)	6	(−4, 15)
Grammaticality	Gender	9	(−10, 27)	**57**	**(42, 76)**	**43**	**(29, 58)**
	Number	**35**	**(13, 60)**	**38**	**(21, 56)**	10	(−5, 24)
**5B: N = 96**							
Attraction ungrammatical	Gender	−4	(−26, 21)	15	(−2, 33)	**16**	**(3, 28)**
	Number	−25	(−58, 3)	−5	(−22, 14)	−1	(−14, 11)
Attraction grammatical	Gender	7	(−18, 30)	−14	(−34, 4)	6	(−5, 19)
	Number	−29	(−53, −6)	−9	(−25, 5)	2	(−11, 16)
Grammaticality	Gender	9	(−25, 41)	**42**	**(19, 67)**	**26**	**(5, 47)**
	Number	**58**	**(24, 95)**	**35**	**(15, 55)**	1	(−22, 22)

*Mean RT for each effect of interest. 95% Confidence Intervals computed by BCa bootstrap (2,000 replications) in parenthesis. Effects in which the CI excludes zero are marked in bold. Effects in which the CI includes zero up to ±1 ms are marked in italic*.

In experiments 5A and 5B alike, a reliable grammaticality effect emerged in the *Verb* region for the number manipulation which continued into the *Verb*+*1* region, whereas a reliable grammaticality effect for gender emerged only in the *Verb*+*1* region and continued into the *Verb+2* region.

When it comes to the gender attraction effects in ungrammatical sentences, they were numerically observed in experiments 5A and 5B at the *Verb*+*1* region (10 and 15 ms), but in neither case the 95% CI excluded 0 (its lower bound included −1 ms in 5A and −2 ms in 5B). The CIs did exclude 0 in the *Verb+2* region (11 ms in 5A and 16 ms in 5B). There was no clear indication of gender attraction effects in grammatical sentences.

In contrast, the results for number attraction effects in ungrammatical sentences were mixed. They were of similar magnitudes compared to the gender attraction effects in experiment 5A in both *Verb* and *Verb*+*1* region (14 and 10 ms, respectively), but in neither case the 95% CI excluded zero (lower bound included −2 ms at the *Verb* region and −3 ms at the *Verb*+*1* region). They were, however, not even numerically observed in 5B: they flipped sign in all critical regions, and in the *Verb* region the 95% CI of experiment 5B [−58, 3] excluded the point estimate of experiment 5A (14 ms), although that was not the case in the *Verb*+*1* region. There was no clear indication of number attraction effects in grammatical sentences.

### 6.5. Discussion

Experiments 5A and 5B provide further support for the notion that gender also participates in illusory agreement, and that it exhibits the grammatical asymmetry that has been described for number in other languages. When it comes to the number attraction effect, experiment 5A and 5B give conflicting results, much like experiments 4A and 4B. Finally, a direct comparison between effect sizes between gender and number in experiments 5A and 5B paint a complex picture. In experiment 5A, gender and number attraction effects in ungrammatical sentences seem roughly equivalent in size, despite their timing difference. In experiment 5B, gender attraction effects seem larger than those of number, but that could be a consequence of the sign shift in the latter. We now turn to sorting out these issues by presenting the results of a meta-analysis of all eight experiments reported here.

## 7. Meta-Analysis

In order to help make sense of the large number of results reported in the preceding eight experiments, we employ a *meta-analysis* (Rosenthal and Dimatteo, 2001; Hunter and Schmidt, 2004; Cumming, 2014; Cooper et al., 2009). In this kind of analysis, we combine the results of multiple experiments testing the same hypothesis into a single joint summary that provides a less biased and better statistically grounded view of the cumulative evidence than just counting whether or not particular experiments exhibited or failed to exhibit the predicted pattern of results.

Here, we opt to conduct a fixed effects instead of a random effects meta-analysis (cf. Cooper et al., 2009), as our analytical goals and pool of experiments all fit the assumptions of the former at a conceptual level. First, our aim is primarily to summarize the results of the eight experiments reported here, and not necessarily extrapolate from them on a statistical basis. Second, the eight experiments reported are either direct replications or extremely similar to each other in terms of their design, procedure, experimental materials, but also in terms of the population being tested—all students from the same university, of similar ages, identical genders, and all tested within a period of 12 months. Finally, the fixed-effect model has the practical advantage of having more power compared to the random effects model (Rosenthal and Dimatteo, 2001).

Our goal is to compare the attraction effects for number and gender, and how they may vary as a function of their timing, effect size and susceptibility to the grammatical asymmetry and the markedness asymmetry. Therefore, we conduct eight meta-analyses on each of the three critical regions we have been focusing on: *Verb, Verb*+*1*, and *Verb+2*. Each analysis is focused on a specific agreement feature (number or gender), a specific grammaticality level (grammatical or ungrammatical) and markedness status (singular/plural or masculine/feminine). In each analysis, the studies were weighed by the inverse of their variance. All analyses were performed using the *metafor* package in the R programming language (Viechtbauer, 2010).

### 7.1. Meta-Analysis of Gender

**Figure 6:**
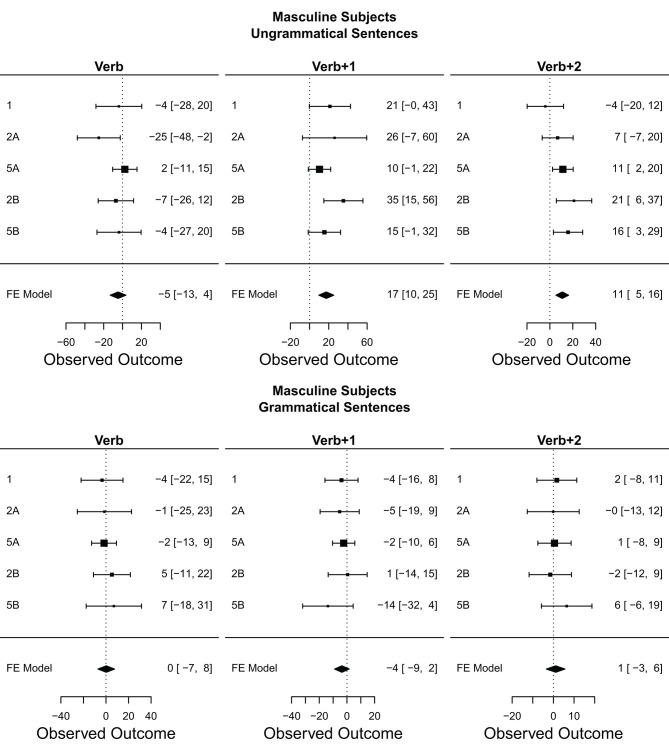
Gender attraction effect in ungrammatical and grammatical sentences: forest plots of meta-analysis for masculine subjects. The size of the square representing each experiment is proportional to its weight in the meta-analysis. The estimated effect size of the meta-analysis is represented by the black diamond at the bottom of each graph. The width of the diamond represents the estimated 95% CI of the meta-analysis effect size. Experiments are plotted in chronological order from top to bottom.

Figure 6 displays the meta-analyses for gender attraction using unmarked (masculine) subjects in ungrammatical vs. grammatical sentences. The results are straightforward: there is a clear grammaticality asymmetry wherein gender attraction only occurs in ungrammatical sentences. Moreover, gender attraction seems to occur in the two regions after the verb. The point-estimate effect size of the effect was 17 ms for the *Verb*+*1* region and 11 ms for the *Verb+2* region, both with meta-analytical 95% CIs excluding zero.

**Figure 7:**
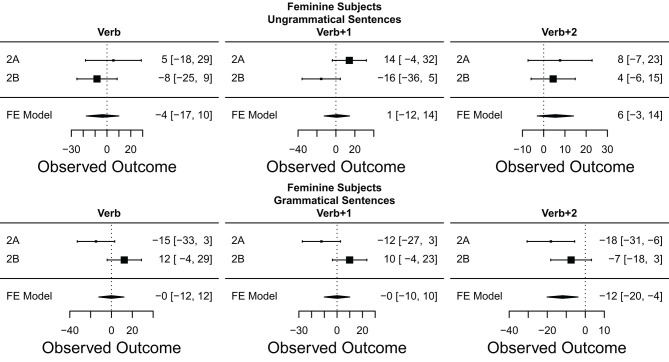
Gender effect in ungrammatical and grammatical sentences: forest plots of meta-analysis for feminine subjects. The size of the square representing each experiment is proportional to its weight in the meta-analysis. The estimated effect size of the meta-analysis is represented by the black diamond at the bottom of each graph. The width of the diamond represents the estimated 95% CI of the meta-analysis effect size. Experiments are plotted in chronological order from top to bottom.

Figure 7 displays the meta-analysis for gender attraction using marked (feminine) subjects in ungrammatical vs. grammatical sentences. Contrary to what has been shown for sentences with unmarked subjects, there is no clear gender attraction effect for sentences with marked subjects, and therefore there cannot be evidence for a grammaticality asymmetry with feminine subjects (Almeida and Tucker, 2017). The only other notable effect is a “reverse” gender attraction effect for grammatical sentences in the *Verb+2* region, a result that unfortunately does not have a clear theoretical interpretation and that we will leave for future research.

The meta-analysis shows clear evidence of a gender attraction effect that is susceptible to the grammatical asymmetry and likely to the markedness asymmetry when the markedness of the subject gender provides the possibility of attraction. This effect is estimated to emerge only in the post-verbal regions, never in the *Verb* region itself.

### 7.2. Meta-Analysis of Number

For the meta-analysis for number attraction effect, we also include the results of Tucker et al. (2015), broken down by their subgroup analysis of sound/suffixing plurals vs. broken/ablauting plurals. The raw data from Tucker et al. (2015) was subjected to the same pre-processing steps as the other eight experiments.

**Figure 8:**
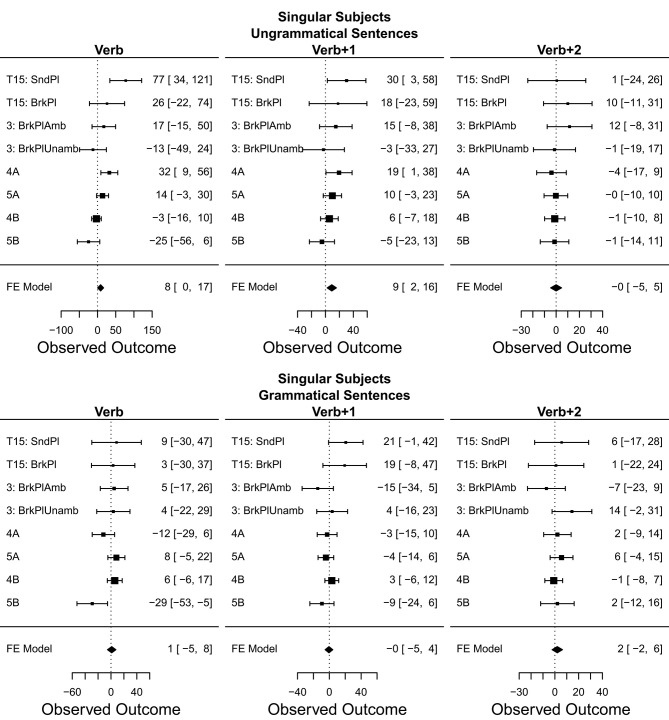
Number effect in ungrammatical and grammatical sentences: forest plots of meta-analysis for singular subjects. The size of the square representing each experiment is proportional to its weight in the meta-analysis. The estimated effect size of the meta-analysis is represented by the black diamond at the bottom of each graph. The width of the diamond represents the estimated 95% CI of the meta-analysis effect size. Experiments are plotted in chronological order from top to bottom.

Figure 8 displays the meta-analysis for number attraction using unmarked (singular) subjects in ungrammatical vs. grammatical sentences. The results show a clear grammaticality asymmetry in that number attraction only occurs in ungrammatical sentences. Moreover, number attraction seems to occur immediately at the *Verb* region as well as its spillover region. The point-estimate effect sizes of the effect were 8 ms for the *Verb* region and 9 ms for the *Verb*+*1* region, both with meta-analytical 95% CIs excluding zero. It should be noted, however, that the lower bound of 95% CI for the attraction effect in the *Verb* region was 0.3 ms.

**Figure 9:**
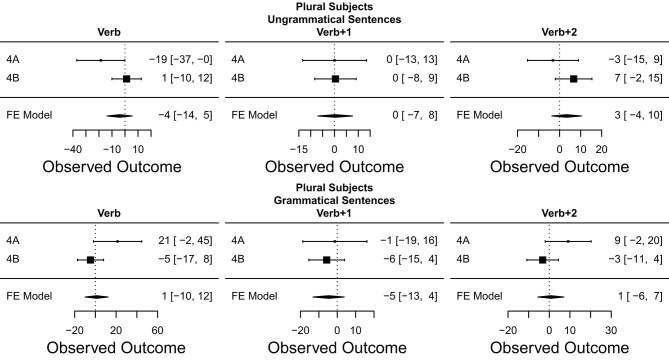
Number effect in ungrammatical and grammatical sentences: forest plots of meta-analysis for plural subjects. The size of the square representing each experiment is proportional to its weight in the meta-analysis. The estimated effect size of the meta-analysis is represented by the black diamond at the bottom of each graph. The width of the diamond represents the estimated 95% CI of the meta-analysis effect size. Experiments are plotted in chronological order from top to bottom.

Figure 9 displays the meta-analysis for number attraction using marked (plural) subjects in ungrammatical vs. grammatical sentences. Contrary to what has been shown for sentences with unmarked subjects, there is no clear number attraction effect for sentences with marked subjects. Thus, there cannot be evidence for a grammaticality asymmetry with plural subjects, as was the case for gender attraction with feminine subjects.

The meta-analysis of number attraction effects included two experiments in which masculine/broken plural attractors were used, namely Tucker et al. (2015) and our experiment 3. Given the uncertainty as to whether these attractors completely fail to induce attraction effects or simply induce smaller ones, it is possible that their inclusion in the meta-analysis artificially depresses the estimate effect size for number attraction. Alternatively, even ignoring the experiments using masculine/broken plural attractors, we observed two experiments with feminine/sound plural attractors which failed to show clear number attraction effects, namely experiments 4B and 5B. In order to better understand the contribution of these two sources of uncertainty, a meta-analysis excluding the two experiments using masculine/broken plural attractors was conducted. However, excluding these experiments did not meaningfully change the estimated attraction effect sizes in the *Verb* and *Verb*+*1* regions, with changes of <0.5 ms in both regions.

The meta-analysis therefore shows evidence of a number attraction effect that is susceptible to the grammatical asymmetry and likely to the markedness asymmetry as well. This effect is estimated to emerge immediately at the verb regions and spills over into the first post-verbal region. Compared to the gender attraction effect, the number attraction effect size is considerably smaller (less than half) when the regions where each effect first emerges is compared (17 ms for gender vs. 8 ms for number). The meta-analytical 95% CI for gender [10, 25] excludes the estimated effect size for number (8 ms), while the 95% CI for number [0, 17], abuts the estimated effect size for gender (17 ms), which is suggestive of an actual difference in magnitude between the two features. The CIs overlap by 7 ms while their average width is 16 ms, leading to a proportion of 44% between the two quantities. According to Cumming and Finch (2005)'s rule-of-thumb for the comparison of independent CIs mentioned earlier, proportions of 50% or lower correspond to *p* < 0.05 in an independent samples *t*-test. Thus, both qualitative and quantitative evidence suggest that there is a real magnitude difference between the gender and number attraction effects in the region each one first occurs.

## 8. General Discussion

The meta-analysis of the eight experiments reported here supports the notion that errors in subject-verb agreement dependency comprehension are, at their core, universal in scope (cf. Lago et al., 2015). Despite the universality of the errors, however, we have uncovered differences between number and gender agreement in comprehension that have ramifications for theories of how long-distance dependencies are processed in real-time.

### 8.1. Dimensions of Similarity

We laid out five criteria as the basis of comparison between the processing of number and gender subject-verb agreement, namely *existence* of attraction effects, their *effect size, time course*, and susceptibility to the *grammatical asymmetry* as well as to the *markedness asymmetry*.

Both gender and number clearly give rise to attraction effects in the comprehension of verbs. In Experiments 1, 2A, 2B, 4A, and 5A, the RT profiles at and immediately following the critical verbs include a facilitation to attraction conditions relative to the large reading time spike seen in response to ungrammatical non-attraction conditions.

Gender and number processing at the verb also exhibit the asymmetry of the attraction effects with respect to the grammatical status of the verb. In all eight experiments reported here, attraction RT profiles, if they are present, are so only in ungrammatical sentences. *Modulo* experiments 3, 4B, and 5B, where no number attraction was detected, throughout all other experiments a difference in the Match vs. NoMatch conditions reliably emerges only when the verb is grammatically disallowed. While there is some contention about the generality of this finding (cf. Franck et al., 2015; Villata et al., 2018; Nicenboim et al., 2018), here we replicate in five experiments the previously reported findings that attraction effects in comprehension do not reliably obtain in grammatical sentences (e.g., Wagers et al., 2009; Dillon et al., 2013; Tanner et al., 2014; Tucker et al., 2015). A possible exception to this generalization could be argued on the basis of the gender attraction results of experiment 2A, in which a “reverse” attraction effect was numerically observed in all critical regions, both in sentences with masculine and feminine subjects, and on the basis of number attraction effect in the *Verb* region for sentences with singular subjects in experiment 5B, another example of a “reverse” attraction effect. However, the effects on experiment 2A were virtually all very small, and suggestive statistical evidence only emerged in the *Verb*+*1* and *Verb+2* regions for sentences with feminine subjects. Moreover, the sign of the effect flipped in the replication experiment 2B. An analogous sign change is observed in the example of 5B: the same estimate in the original experiment 5A was in the other direction. Finally, no statistically suggestive evidence is obtained in the meta-analysis when the results of the replications (2B and 5A) are combined, nor in the larger analysis of gender and number attraction effects in grammatical sentences. The equivalency (or lack thereof) of attraction effects in both grammatical and ungrammatical sentences is an empirical point of distinction between competing theories of attraction effects—cue-based memory retrieval theories are arguably better-equipped to handle these asymmetries than misrepresentation theories, a point to which we return below, and thus it is important to determine whether observable attraction effects in grammatical sentences in individual experiments are reliable or seem spurious. Here we believe the weight of the evidence favors the latter interpretation.

Another similarity observed for gender and number across our experiments has to do with the presence of the asymmetry that we have been calling markedness-based. In MSA, plural number is marked (in the sense of Trubetskoy, 1939/1958) relative to singular and feminine gender is marked relative to masculine. If gender and number are equivalent along the markedness dimension and in line with the markedness results reported for English (Bock and Miller, 1991; Eberhard, 1997), one would expect that attraction effects to be present and/or strongest for singular subjects with plural attractors and masculine subjects with feminine attractors. In contrast, one would expect attraction effects to be absent or greatly reduced for plural subjects with singular attractors and feminine subjects with masculine attractors. Here we find no evidence for attraction effects when subjects carry a marked agreement feature, either for ungrammatical or grammatical sentences, while the evidence of attraction when the subjects carry an unmarked agreement feature is substantial.

Despite all these similarities between the process of gender and number verbal agreement, we nonetheless also observed important differences between them. There is a clear effect size difference in attraction effects of number and gender: in the region where they first emerge, the former is half the size of the latter (8 vs. 17 ms), but they seem to align in their respective spillover regions (9 vs. 11 ms). Interestingly, our estimated effect size for the first manifestation of gender attraction in the meta-analysis (17 ms) is quite close to the 22 ms effect size that has been estimated in a recent meta-analysis of number attraction effects (Jäger et al., 2017). Crucially, the 95% CI for this effect (95% CI [10, 25]) actually excludes the point estimate of the first manifestation of the number attraction effect observed (8 ms), as does the 95% Credible Interval reported by Jäger et al. (2017) (95% CrI [9, 36]), indicating that number attraction in Arabic does appear to differ from what is observed in other languages.

Finally, we also observe that the time course of attraction effects varies between number and gender agreement. Although the nature of the self-paced reading methodology employed in this study is suboptimal to fully resolve this issue, our results consistently indicate that gender attraction effects emerge at the *Verb*+*1* region, while the number attraction effect emerges systematically at the *Verb* region whenever it is found (see also Figures 6, 8). Interestingly, in three out of five experiments the gender attraction effect occurs in the region following the one where the grammaticality effect first show strong evidence of occurring, in stark contrast with the number attraction effect, which always occurs as soon as the grammaticality effect is detected. This finding is especially relevant given the recent observations by Lago et al. (2015) that attraction effects can, in principle, appear after grammaticality effects in self-paced reading data.

### 8.2. Implications for the Representation and Processing of Agreement

Given the importance of representational commitments in theories of online long distance dependency processing, it is crucial to consider whether our results could be accounted for in ways neutral to psycholinguistic theories if simple changes in how linguistic features are used in processing or mapped onto cues for memory retrieval are considered. Here we entertain two approaches to featural representation: (1) an approach which localizes the difference in the valency of feature representation (i.e., Fuchs et al., 2015) and (2) one which localizes the difference in the location of gender information in grammar and processing (i.e., Deutsch and Dank, 2011).

One approach to asymmetries between gender and number would be to assert that these features are simply represented differently in grammar or processing. For instance, one could follow the approach of Fuchs et al. (2015) and assert that agreement features which show markedness asymmetries are privative—they are represented only in the marked value and not present otherwise. Features which do not show markedness contrasts are instead equipollent—they are represented by the presence of features regardless of markedness. Fuchs et al. (2015), use this idea to represent the differential activity of gender and number in Spanish agreement attraction, and one could extend it to Arabic by positing that gender is bivalent ([±Masc]) whereas number is privative ([Pl] or ∅). From this assumption one could tie either misrepresentation or cue-based retrieval models to this featural specification.

The problem with this approach is that it is not sufficiently supported by the distributional properties of the MSA grammar. For one, equipollent featural representations are typically used to encode three-way contrasts, which gender is not in Arabic—there is no neuter gender in MSA. While this is not an insurmountable representational issue, it does mean that the only evidence for equipollent gender in MSA would be the very markedness patterns that must be explained. A larger issue, however, has to do with number. Grammatical number in MSA is not a two-way system, but instead a three-way system, including a morphological dual which is used for sets of cardinality two (Ryding, 2005). Three-way distinctions are more difficult to encode in privative feature systems since privative representations are meant to encode two-way contrasts. What is needed to properly assess this question is a comparison of our results concerning singular and plural number with similar data concerning the dual in MSA (but see Ristic et al., 2016 for evidence of markedness asymmetries even in the tripartite number system of Serbian).

A different approach to these issues would be to assert that gender and number are represented in different components of the processing system: one of these features is inherent to lexical meaning whereas the other is not. By way of example, Deutsch and Dank (2011) suggest that one could capture an identical pattern to our results but for Hebrew gender and number production data by assuming that gender is an inherent property of the lexical entry of a noun and not part of the morphophonological properties of the word supplied by its structural or morphological context (see also Sicuro Corrêa et al., 2004; Carminati, 2005 for similar ideas in the psycholinguistic literature based on Romance languages, as well as theoretical proposals wherein gender is instantiated lower in a nominal's structural representation, as in Kramer, 2009). Grammatical number, on the other hand, is not an inherent property of words, since any given lexical entry can be either singular or plural from a conceptual standpoint. Embedded most comfortably in a representational account of attraction, this would mean that an NP containing an attractor can have properties which are not part of its lexical entry (such as number) more easily overwritten or confused than properties which are intrinsic (such as gender).

However, while this approach is very well-suited to gating the presence or absence of attraction, it is incapable of attenuating or strengthening attraction effects in similar dimensions. Our results suggest that gender and number are qualitatively alike from an attraction standpoint, yet quantitatively distinct, with gender being a later, larger attraction process than number. If intrinsic lexical properties are robust to attraction, they should be less confusable in comprehension—this will be true regardless of the specific model implementation. Even if a version of this model could be sketched which could predict qualitative identity between gender and number, the existence of a quantitative asymmetry remains an issue, since the putatively inherent and robust feature (gender) is susceptible to stronger, not weaker, attraction effects compared to the non-lexically determined feature (number). More generally, one can step back and see that any attempt to explain our results based upon the representational structure or geometry of the features involved will be incapable of explaining the quantitative results we have observed in this study, geared as they are toward explaining the presence or absence—not quantity—of attraction.

Given that a simple representational change is not sufficient for explaining the differential effects that we observe for agreement attraction with gender and number, we now return to the two major classes of theories discussed in the introduction in light of these results. While both kinds of theories require non-trivial changes to their architectures to account for differences between gender and number, we ultimately suggest that cue-based retrieval theories require less drastic modifications (i.e., such as those proposed in Engelmann et al., 2019).

Our results present two major challenges for misrepresentation theories broadly speaking: (1) the differential quantitative strength of gender and number attraction and (2) the absence of agreement attraction in grammatical sentences. Both of these challenges stem from a similar prediction common to representational theories: since theories that attribute attraction effects to failures of representation take the agreement process itself to be undisturbed when attraction occurs, they predict qualitative and quantitative parity of attraction effects across identically represented subjects. What causes attraction in, e.g., the theories of Eberhard et al. (2005), Franck et al. (2002), Nicol et al. (1997), and Vigliocco and Nicol (1998) is a process by which structural representations of the subject are malleable enough to allow features of the attractor to be copied erroneously to the verb by the normal processes of subject-verb agreement. It is a corollary of this assumption that attraction should occur in equal measure in structurally identical subject noun phrases (Wagers et al., 2009). However, this is not what we observe in this study. Our results suggest weaker attraction effects for number in MSA than for gender. Given that our experiments involved structurally identical subject and attractor NPs across all experiments, these results cannot be explained by reference to different structural configurations leaking attractor features in different strengths. Number attraction appears diminished in strength relative to gender when compared directly in a subject relative clause configuration in both cases.

To account for these results, the Marking and Morphing Model of Bock et al. (2001) and Eberhard et al. (2005) could attempt to derive them from our use of animate human-denoting nouns in all four experiments. In the Marking and Morphing Model, attraction occurs during one of two processes: (1) marking, responsible for translating message-level conceptual information into a grammatical representation and (2) morphing, responsible for unifying and reconciling features of the subject and any conflicting morphosyntactic features present in attractors. Marking is argued to be responsible for notional attraction effects, such as more attraction when a distributive interpretation is possible, whereas morphing is responsible for morphosyntactic effects, such as the susceptibility of singular nouns to agree as though they were plural when a constrained attractor has an unambiguously plural suffix. Since both gender and number contain the same morphological instantiation in our data, a suffix in the marked case which is not present in the unmarked case, morphing is unavailable as an explanation for asymmetric attraction effects. Marking is similarly problematic, however, as all of our stimuli contained animate, human-denoting nouns where grammatical gender is conceptually meaningful. This means that gender on a subject noun phrase should be robust to interference from subordinate attractors with mismatching gender, given that the subject already has a notional gender which cannot be easily discarded. This should in turn predict a lack of attraction or greatly attenuated attraction effect for gender, contrary to what we find here.

More broadly, however, both quantitative and qualitative misrepresentation models (including the Marking and Morphing Model) struggle with the lack of attraction consistently observed in our studies in grammatical sentences. As Wagers et al. (2009) have argued, these models cannot predict anything other than quantitative parity in the rates of attraction, since the malleable or leaky representation of subjects occurs blind to what happens at the verb. This point holds even if one accepts the existence of very small grammatical agreement attraction effects (Franck et al., 2015; Villata et al., 2018; Nicenboim et al., 2018), as we have shown a large quantitative difference which cannot be accounted for under misrepresentation approaches. The only misrepresentation approach which could account for these sorts of effects is the degraded memory representation model of Staub (2009, 2010), though this model too needs modifications to successfully predict differential strengths of attraction for number and gender. Further work is required to account for this quantitative asymmetry in misrepresentation models as they are presently understood.

However, recent work by Hammerly et al. (2019) has challenged the notion that grammatical asymmetry effects cannot be captured by misrepresentation models, such as Marking and Morphing. They show that, in presence of response bias, such models could indeed predict grammatical asymmetry effects in explicit decision tasks, such as speeded acceptability judgment tasks. Furthermore, Hammerly et al. (2019) also show that when response bias is minimized or eliminated, so are the grammaticality asymmetries found in speeded acceptability judgment tasks. While it is possible that such an account could apply to results like ours, it is not immediately obvious how it would. Hammerly et al. (2019) modeled and tested the influence of response bias in terms of a forced binary response in a highly meta-linguistic task. In our experiment, however, participants engaged in self-paced reading purely for comprehension. The only binary decision they were required to make was to judge a subsequent comprehension question as true or false. These questions never referred to the agreement status of the verb. Moreover, the RT to this decision was not the dependent measure of the study either. In the absence of further refinement, it is difficult to assess whether the interesting proposal put forth by Hammerly et al. (2019) can actually be applied to more implicit, less clearly decisional measures, such as self-paced reading, eye-tracking and ERP results, but this clearly constitutes an interesting topic for future research.

Cue-based retrieval theories, on the other hand, deal much more successfully with the lack of attraction in grammatical sentences. In these models (such as those deriving from Lewis and Vasishth, 2005 and Badecker and Lewis, 2007), attraction occurs when cue-mismatches between subjects and attractors lead to the erroneous retrieval of the attractor during a working memory retrieval event triggered by the verb. There are two distinct ways to concretize this idea: either the retrieval event occurs in all instances or it only occurs upon the presentation of ungrammatical verbs. In either case, however, grammatical attraction is not predicted.

What is less obviously representable in these models is our finding of a quantitative asymmetry between gender and number attraction, though we suggest below that revisions are possible to account for this fact. All cue-based retrieval models are dependent upon the exact cue structure assumed, and in the first versions of these models (Lewis and Vasishth, 2005; Badecker and Lewis, 2007) the cue structures posited would not predict quantitative asymmetries. In the model of Lewis and Vasishth (2005), for example, the strength of a cue can only be modulated as a function of the number of other cues in memory (“the fan”), not the intrinsic properties of the cue itself. Thus, gender could be strong relative to number, but only if number uniformly occurred as a cue in contexts where more cues were available in the system in general. Obviously, this is not a feasible assumption for MSA, where gender and number always co-occur on verbs (Ryding, 2005).

However, recent innovations in these models have stressed the importance of cue weighting in describing the relative inability of many researchers to find attraction effects with reflexive-antecedent dependencies (Dillon et al., 2013; Cunnings and Sturt, 2014; Parker and Phillips, 2017). While cue-weighting has always been an assumed feature of these models, early work (such as Lewis and Vasishth, 2005) took all cues to be equally weighted. In cue-based retrieval models, the amount of activation which a matching goal receives from a particular cue is a function of that cue's weight in the retrieval system. Parker and Phillips (2017) suggest that structural cues (such as appearing in the structurally appropriate place for a subject) must be weighted more heavily in the retrieval of reflexive antecedents than morphological cues (such as being grammatically feminine). This drives down the impact of morphological mismatches and in some cases eliminates attraction altogether, leading to the mixed results in the literature concerning whether or not reflexive-antecedent dependencies are subject to the same interference as subject-verb agreement. Following this reasoning, one could postulate that cues for number are less strongly weighted than those for gender. When a verb is ungrammatical for reasons of number morphology, a distractor which matches the verb in number cues would receive a smaller activation boost relative to a distractor which matches a verb in gender cues when the verb is ungrammatical for reasons of gender morphology. This would predict that the size of the attraction effect and number of erroneous retrievals should be smaller in the case of number attraction than gender attraction, exactly as we observe in our data. We tentatively suggest that this is the case, pending explicit computational modeling of this idea in future work.

Finally, it is worth noting that neither misrepresentation nor cue-based retrieval models could account for differences in timing of gender and number attraction effects. Attraction is a verbal process, meaning that the representations and processes responsible for these effects should be keyed at the verb, not later. As we observed, it is possible that our evidence hints at the delayed appearance of attraction for gender relative to attraction for number. Self-paced reading methodologies commonly involve spillover effects with no clear theoretical explanation, but even when taking these into account, the combined data from our eight experiments strongly suggests a *Verb* locus for the number attraction effect and a *Verb*+*1* locus for the gender attraction effect. More research using methods with better time resolution than the self-paced reading paradigm should be performed in MSA to check the robustness of this differential timing effect. In addition, more research is needed on cue-based retrieval models to incorporate this differential timing, should it be prove to be robust.

### 8.3. Conclusions

We have demonstrated that subject-verb gender agreement attraction occurs in comprehension. Moreover, these results obtain in an inflectionally rich language in relative clause configurations where attraction should be smaller in effect, all else equal. We have also demonstrated that attraction for gender and number is not identical in Arabic. Quantitatively, we demonstrated that agreement attraction for gender is stronger relative to number attraction but occurs later in time. We also added additional evidence to the body of work suggesting that comprehension attraction effects do not occur in grammatical sentences, for gender or number. These results were argued to be largely more compatible with cue-based retrieval models over misrepresentation models insofar as the former are capable of accounting for grammaticality asymmetries and require fewer alterations to account for quantitative differences among agreement features. We suggested that much progress can be made in theorizing about attraction by moving from simply establishing the presence or absence of agreement attraction patterns to work using large sample sizes on other cues and languages that focuses on direct comparisons of agreement features.

At a methodological level, we note that despite employing relatively large sample sizes, we still observed numerous apparent failures to replicate individual effects across the studies we reported (in particular number attraction, see Figure 8). This should alert psycholinguists used to running smaller experiments and not conducting systematic replications of their own work that our field's expectations about the replicability of findings of individual studies is often too optimistic (Vasishth et al., 2018), but that nonetheless firmer conclusions can be reached and justified by using meta-analytical techniques.

## Data Availability Statement

The link to the data and analysis scripts is: https://figshare.com/projects/Attraction_Effects_for_Verbal_Gender_and_Number_Are_Similar_but_Not_Identical_Self-Paced_Reading_Evidence_from_Modern_Standard_Arabic/18823.

## Ethics Statement

The studies involving human participants were reviewed and approved by New York University Abu Dhabi Institutional Review Board (IRB). The patients/participants provided their written informed consent to participate in this study.

## Author Contributions

MT, AI, and DA designed the experiments, discussed the results, and wrote the paper. MT and AI oversaw the creation of the materials. MT and DA oversaw the collection of the data and performed the data analysis. All of MT's work on this manuscript was completed before his employment at Amazon.com, Inc. All authors contributed to the article and approved the submitted version.

## Conflict of Interest

The authors declare that the research was conducted in the absence of any commercial or financial relationships that could be construed as a potential conflict of interest. All of MT's work on this manuscript was completed before his employment at Amazon.com, Inc.

## Footnotes

^1^Here the *(correct) subject* appears in italic face, the **attractor/distractor** NP in bold face, and the ***target region***in both bold and italic.

^2^While there is a considerable literature on noun-adjective gender agreement (e.g., Vigliocco and Franck, 1999; Vigliocco and Zilli, 1999; Vigliocco and Franck, 2001; Antón-Méndez et al., 2002; Franck et al., 2008; Deutsch and Dank, 2009; Dank and Deutsch, 2010; Deutsch and Dank, 2011; Acuña-Fariña et al., 2014; Fuchs et al., 2015; Alemán Bañón and Rothman, 2016; Paspali and Marinis, 2020) and its contrast with number agreement in the nominal domain (e.g., Bañón et al., 2012; Barber and Carreiras, 2005; Popov and Bastiaanse, 2018), in addition to some recent investigations on noun-clitic agreement (Santesteban et al., 2017; Paspali and Marinis, 2020), we focus here on *subject-verb* agreement, since it is not necessarily the case that these other type of dependencies are underwritten by identical mechanisms—crucially, noun-adjective and determiner-noun agreement are not potentially unbounded in the way verbal agreement is. (See Baker, 2008 and Norris, 2014). Moreover, with few notable exceptions, these studies largely examine production, which is not equivalent to comprehension in agreement attraction studies (Tanner et al., 2014).

^3^The gender composition of our participant sample is due to the nature of instruction at the UAEU—there are separate campuses for male and female students, and all participant testing was conducted on the female campus.
