# Supplementary material for: Attraction Effects for Verbal Gender and Number Are Similar but Not Identical: Self-Paced Reading Evidence From Modern Standard Arabic
Source: Front Psychol. 2021 Jan 21;11:586464. doi: 10.3389/fpsyg.2020.586464 (PMC7859339; doi:10.3389/fpsyg.2020.586464)
Supplement: Supplementary file 1 [file Data_Sheet_1.pdf]

Supplementary Material for: Attraction Effects for Verbal Gender and Number Are Similar but Not Identical: Self-Paced Reading Evidence from Modern Standard Arabic

Matthew A. Tucker Ali Idrissi Diogo Almeida

Contents

1 Complete Materials – Experiments 1–2 2

2 Complete Materials — Experiment 3 5

3 Complete Materials — Experiment 4 9

4 Complete Materials — Experiment 5 13

1 Complete Materials – Experiments 1–2

A.1. The translator who helped the manager occasionally speaks five languages fluently.
المترجم الذي ساعد المدير أحياناً يتكلم خمس لغات بفصاحة.

A.2. The student who saw the professor yesterday studied electrical engineering at the university.
الطالب الذي رأى الأستاذ بالأمسِ درس الهندسة الكهربائية في الجامعة.

A.3. The engineer who met the scientist by chance is working on a new invention.
المهندس الذي استقبل العالِم بالصدفةِ يعمل على ابتكار جديد.

A.4. The cook who scolded the waiter forcefully works in an expensive restaurant during the summer.
الطباخ الذي وبّخ النادل بشدةٍ يشتغل في مطعم غالٍ خلال الصيف.

A.5. The analyst who advised the minister intelligently discusses the Palestinian issue in depth.
المحلل الذي نصح الوزير بذكاءٍ يتناول القضية الفلسطينية بعمق.

A.6. The child who saw the prince before visits the royal family each week.
الطفل الذي رأى الأمير سلفاً يزور العائلة الملكية كل أسبوع.

A.7. The teacher who taught the child dedicatedly attended the graduation party of the students.
المعلم الذي علّم الطفل بتفانٍ حضر حفل تخرج الطلاب.

A.8. The consultant who warned the president yesterday found a solution for the financial problem.
المستشار الذي حذّر الرئيس بالأمسِ وجد الحل للمشكلة المالية.

A.9. The driver who accompanied the ambassador regularly works seven days a week.
السائق الذي رافق السفير بانتظامٍ يعمل سبعة أيام في الأسبوع

A.10. The jailor who tortured the prisoner constantly cleans the cells nightly.
السجّان الذي عذّب السجين باستمرارٍ ينظف الزنازين كل ليلة.

A.11. The broadcaster who talked to the activist yesterday trained in a famous company.
المذيع الذي كلم الناشط بالأمس تدرب في شركة مشهورة.

A.12. The employee who helped the colleague humbly gained the confidence of all the colleagues at work.
الموظف الذي ساعد الزميل بتواضع استطاع كسب ثقة باقي الزملاء في العمل.

A.13. The singer who challenged the poet arrogantly has lost for not showing up on time.
المنشد الذي تحدى الشاعر بغرور خسر لعدم حضوره في الوقت المحدد.

A.14. The beginner who questioned the expert daily has acquired good experience.
المبتدئ الذي سأل الخبير يومياً اكتسب خبرة جيدة.

A.15. The man who hosted the friend with pleasure slept in the basement of the house.
الرجل الذي استضاف الصديق بسرور نام في الطابق السفلي من المنزل.

A.16. The patient who consulted the doctor yesterday returned home satisfied.
المريض الذي استشار الطبيب بالأمس عاد الى المنزل مطمئنا.

A.17. The teacher who met the writer happily likes reading about literature.

المعلم الذي استقبل الأديب بسعادةٍ يحب القراءة عن الأدب.

A.18. The manager who phoned the partner in the morning plans to expand the branches of the company.

المدير الذي هاتف الشريك بالصباح يخطط لتوسيع فروع الشركة.

A.19. The chef who invited the guest in the evening masters preparing various delicious dishes.

الطاهي الذي دعا النزيل بالمساء يتقن إعداد أصناف لذيذة من الطعام.

A.20. The child who saw the magician with amazement applauded hysterically during the show.

الطفل الذي رأى الساحر بانبهار صفق بشدة خلال العرض.

A.21. The young man who helped the wounded man in the morning spends the whole week reading.

الشاب الذي ساعد الجريح صباحاً يقضي كل الأسبوع في المطالعة.

A.22. The doctor who healed the captive quickly is joining the national guard (army).

الطبيب الذي عالج الأسير بسرعةٍ ينخرط في فرقة الدفاع المدني.

A.23. The ruler who imprisoned the criminal previously practiced justice and equality among the people.

الحاكم الذي سجن المجرم سابقاً مارس العدل والمساواة على الشعب.

A.24. The coach who trained the partner efficiently possesses more than 10-years of experience.

المدرب الذي درّب الرفيق بإتقان يمتلك خبرة تفوق العشر سنين.

A.25. The Imam who advised the neighbor wisely lives in the local neighborhood.

الإمام الذي نصح الجار بحكمةٍ يعيش في المنطقة المجاورة.

A.26. The policeman who questioned the murderer seriously asked the pedestrians the reasons for the crime.

الشرطي الذي استجوب القاتل بجديّةٍ سأل المشاة عن سبب الجريمة.

A.27. The lawyer who called the inheritor in the morning discussed the issue of the inheritance distribution.

المحامي الذي استدعى الوريث بالصباح ناقش موضوع تقسيم الإرث.

A.28. The teacher who taught the student well worked as a host in television.

الأستاذ الذي علّم الطالب جيداً عمل كمذيع في التلفزيون.

A.29. The immigrant who spoke with the visitor for a long time feels nostalgic for the country always.

المهاجر الذي حدّث الزائر طويلاً يشعر بالحنين للوطن دائماً.

A.30. The journalist who interviewed the leader persistently published the article in the national newspaper.

الصحافي الذي استجوب الحاكم بالحاح نشر المقال في الجريدة الوطنية.

A.31. The pilot who greeted the airline attendant warmly asked many questions during the journey.

الطيار الذي حيّا المضيف بحرارةٍ سأل أسئلة كثيرة خلال الرحلة.

A.32. The man who talked to the monk intelligently works in the post office.
الرجل الذي كلم الراهب بفطنةٍ يشتغل في مكتب البريد.

A.33. The policeman who arrested the driver quickly helps the pedestrians in crossing the street.
الشرطي الذي اعتقل السائق بسرعةٍ يساعد المارة على عبور الشارع.

A.34. The lawyer who startled the witness cunningly stopped the accusation of his client in the court.
المحامي الذي أربك الشاهد بدهاءٍ أبعد التهم عن موكله في المحكمة.

A.35. The worker who helped the soldier yesterday drives a large truck for the company.
العامل الذي ساعد الجندي بالأمسِ يَسوق شاحنة كبيرة لفائدة الشركة.

A.36. The artist who served the king devotedly gave a portrait to the ambassador of the United States.
الفنان الذي خدم الملك بتفانٍ تبرع بلوحة لسفير الولايات المتحدة.

A.37. The journalist who hosted the star brilliantly raised very embarrassing questions.
الإعلامي الذي استضاف النجم بتألق طرح أسئلة محرجة جداً.

A.38. The nurse who treated the patient carefully studies at the university hospital.
الممرض الذي عالج المريض بعنايةٍ يدرس في مستشفى الجامعة.

A.39. The seller who thanked the customer enthusiastically was happy with the large profit.
البائع الذي شكر الزبون بحماسٍ فرح بالفائدة الكبيرة.

A.40. The coach who was very interested in the player worked at the National Fencing Academy.
المدرب الذي اهتم باللاعب جداً اشتغل في الأكاديمية الوطنية للمبارزة.

A.41. The soldier who met the policewoman yesterday loved the atmosphere at the air base.
الجندي الذي قابل الشرطي بالأمسِ أحبّ العمل في القاعدة الجوية.

A.42. The singer who met the dancer previously sings with the city orchestra.
المطرب الذي استقبل الراقص سابقاً يغني مع أوركسترا المدينة.

A.43. The producer who enthusiastically chose the actor produces a film every month.
المخرج الذي اختار الممثل بشغفٍ ينتج فيلما كل شهر.

A.44. The servant who helped the caregiver earnestly cleans the rooms seven days a week.
الخادم الذي ساعد المربي بجدٍّ ينظف الغرف سبعة أيام في الأسبوع.

A.45. The announcer who invited the historian nicely presented a program on TV.
المذيع الذي دعا المؤرّخ بلطفٍ قدم برنامجاً في التلفزيون.

A.46. The assistant who served the pharmacist loyally collected all of the old reports.
المساعِد الذي خدم الصيدلانيّ بإخلاصٍ جمع كل التقارير القديمة.

A.47. The journalist who interviewed the winner by chance writes in many newspapers.
الصحفي الذي قابل الفائز صدفةً يكتب في صحف كثيرة.

A.48. The bedouin who visited the farmer at night lives in the middle of the desert.
البدوي الذي زار المُزارع ليلاً يسكن في وسط الصحراء.

2 Complete Materials — Experiment 3

Ambiguous	Unambiguous
$C_1aC_2aC_3a$	$C_1uC_2aC_3aa$ʔ
$C_1aC_2iiC_3$	ʔ$aC_1aaC_2iC_3a$
$C_1iC_2aaC_3$	$C_1awaaC_2iC_3$
$C_1iC_2C_3aan$	ʔ$aC_1C_2aaC_3$
$C_1uC_2C_3aan$	ʔ$aC_1C_2iC_3aa$ʔ
$C_1aC_2C_3aa$	
$C_1uC_2C_2aaC_3$	
$C_1uC_2uuC_3$	

Table 1: Templates and ambiguity assignments for broken plural templates in Experiment 3.

B.1. The child who saw the magician with amazement applauded hysterically during the show.
(السحرة) الطفل الذي رأى الساحر بانبهار صفق بشدة خلال العرض.

B.2. The investigator who grabbed the robber at night installs listening devices everywhere.
(الخونة) المحقق الذي أمسك الخائن بالليل يضع أجهزة تنصت في كل مكان.

B.3. The young man who helped the wounded man in the morning spends the whole week reading.
(الجرحى) الشاب الذي ساعد الجريح صباحاً يقضي كل الأسبوع في المطالعة.

B.4. The doctor who healed the captive quickly is joining the national guard (army).
(الأسرى) الطبيب الذي عالج الأسير بسرعةٍ ينخرط في فرقة الدفاع المدني.

B.5. The ruler who freed the slave in the past practiced justice and equality among the people.
(العبيد) الحاكم الذي أعتق العبد قديمًا مارس العدل والمساواة على الشعب.

B.6. The driver who transported the pilgrim at noon drove the car very quickly.
(الحجيج) السائق الذي أخذ الحاج بالظهيرةِ قاد السيارة بسرعة فائقة.

B.7. The carpenter who employed the man regularly made wonderful furniture for the exhibition.
(الرجال) النجار الذي شغّل الرجل بانتظام أعدّ قطع أثاث رائعة للمعرض.

B.8. The coach who trained the partner efficiently possesses more than 10-years of experience.
(الرفاق) المدرب الذي درّب الرفيق بإتقان يمتلك خبرة تفوق العشر سنين.

B.9. The tv-host who presented the bridegroom skillfully met with the minister at the New Year's party.
(العرسان) المذيع الذي قدّم العريس ببراعةٍ قابل الوزير في حفل رأس السنة.

B.10. The Imam who advised the neighbor wisely lives in the local neighborhood.
(الجيران) الإمام الذي نصح الجار بحكمةٍ يعيش في المنطقة المجاورة.

B.11. The policeman who questioned the murderer seriously asked the pedestrians about the reasons for the crime.
(القتلة) الشرطي الذي استجوب القاتل بجدّيّةٍ سأل المشاة عن سبب الجريمة.

B.12. The lawyer who called the inheritor in the morning discussed the issue of the inheritance distribution.

(الورثة) المحامي الذي استدعى الوريث بالصباح ناقش موضوع تقسيم الإرث.

B.13. The teacher who taught the student well worked as a host in television.

(الطلاب) الأستاذ الذي علّم الطالب جيداً عمل كمذيع في التلفزيون.

B.14. The businessman who trained the workers frequently learned English in the UK.

(العمال) التاجر الذي درّب العامل كثيراً تعلم اللغة الانجليزية في بريطانيا.

B.15. The (football) player who opposed the referee angrily won the prize of best player.

(الحكام) اللاعب الذي عارض الحَكم بغضبٍ فاز بجائزة أفضل لاعب.

B.16. The immigrant who spoke with the visitor for a long time feels nostalgic for the country always.

(الزوار) المهاجر الذي حدّث الزائر طويلاً يشعر بالحنين للوطن دائماً.

B.17. The journalist who interviewed the leader persistently publishes the article in the national newspaper.

(الحكام) الصحافي الذي استجوب الحاكم بالحاح نشر المقال في الجريدة الوطنية.

B.18. The pilot who greeted the knight warmly asked many questions during the journey.

(الفرسان) الطيار الذي حيّا الفارس بحرارةٍ سأل أسئلة كثيرة خلال الرحلة.

B.19. The man who talked to the monk intelligently works in the post office.

(الرهبان) الرجل الذي كلم الراهب بفطنةٍ يشتغل في مكتب البريد.

B.20. The policeman who arrested the thief quickly helps the pedestrians in crossing the street.

(اللصوص) الشرطي الذي اعتقل اللص بسرعةٍ يساعد المارة على عبور الشارع.

B.21. The lawyer who startled the witness cunningly stopped the accusation of his client in the court.

(الشهود) المحامي الذي أربك الشاهد بدهاءٍ أبعد التهم عن موكله في المحكمة.

B.22. The worker who helped the soldier yesterday drives a large truck for the company.

(الجنود) العامل الذي ساعد الجندي بالأمسِ يَسوق شاحنة كبيرة لفائدة الشركة.

B.23. The artist who served the king devotedly gave a portrait to the ambassador of the United States.

(الملوك) الفنان الذي خدم الملك بتفانٍ تبرع بلوحة لسفير الولايات المتحدة.

B.24. The journalist who hosted the star brilliantly raised very embarrassing questions.

(النجوم) الإعلامي الذي استضاف النجم بتألق طرح أسئلة محرجة جداً.

B.25. The translator who worked for the manager occasionally speaks five languages fluently.

(المدراء) المترجم الذي ساعد المدير أحياناً يتكلم خمس لغات بفصاحة.

B.26. The student who saw the professor yesterday studied electrical engineering at the university.

(الأساتذة) الطالب الذي رأى الأستاذ بالأمسِ درس الهندسة الكهربائية في الجامعة.

B.27. The engineer who met the scientist by chance is working on a new invention.

(العلماء) المهندس الذي استقبل العالِم بالصدفةِ يعمل على ابتكار جديد.

B.28. The cook who scolded the waiter forcefully works in an expensive restaurant during the summer.

(النوادل) الطباخ الذي وبّخ النادل بشدةٍ يشتغل في مطعم غالٍ خلال الصيف.

B.29. The analyst who advised the minister intelligently discusses the Palestinian issue in depth.
(الوزراء) المحلل الذي نصح الوزير بذكاءٍ يتناول القضية الفلسطينية بعمق.

B.30. The child who saw the prince before visits the royal family each week.
(الأمراء) الطفل الذي رأى الأمير سلفاً يزور العائلة الملكية كل أسبوع.

B.31. The teacher who taught the child dedicatedly attended the graduation party of the students.
(الأطفال) المعلم الذي علّم الطفل بتفانٍ حضر حفل تخرج الطلاب.

B.32. The criminal who attacked the boy viciously breaks through the checkpoint every night.
(الأولاد) المجرم الذي هاجم الولد بشراسةٍ يخترق نقطة التفتيش كل ليلة.

B.33. The consultant who warned the president yesterday found a solution for the financial problem.
(الرؤساء) المستشار الذي حذّر الرئيس بالأمسِ وجد الحل للمشكلة المالية.

B.34. The driver who accompanied the ambassador regularly works seven days a week.
(السفراء) السائق الذي رافق السفير بانتظامٍ يعمل سبعة أيام في الأسبوع.

B.35. The jailor who tortured the prisoner constantly cleans the cells nightly.
(السجناء) السجّان الذي عذّب السجين باستمرارٍ ينظف الزنازين كل ليلة.

B.36. The broadcaster who talked to the activist yesterday trained in a famous company.
(النشطاء) المذيع الذي كلم الناشط بالأمس تدرب في شركة مشهورة.

B.37. The employee who helped the colleague humbly gained the confidence of all the colleagues at work.
(الزملاء) الموظف الذي ساعد الزميل بتواضع استطاع كسب ثقة باقي الزملاء في العمل.

B.38. The singer who challenged the poet arrogantly has lost for not showing up on time.
(الشعراء) المنشد الذي تحدى الشاعر بغرور خسر لعدم حضوره في الوقت المحدد.

B.39. The man who consulted the forgiver yesterday wants retribution of sin/guilt.
(الشفعاء) الرجل الذي استشار الشفيع البارحة يريد التكفير عن الذنب.

B.40. The old man who has mischievously insulted the scholar strives to create problems.
(الفقهاء) العجوز الذي أهان الفقيه بخبث يسعى الى افتعال المشاكل.

B.41. The beginner who questioned the expert daily has acquired good experience.
(الخُبراء) المبتدئ الذي سأل الخبير يومياً اكتسب خبرة جيدة.

B.42. The man who hosted the friend with pleasure slept in the basement of the house.
(الأصدقاء) الرجل الذي استضاف الصديق بسرورٍ نام في الطابق السفلي من المنزل.

B.43. The patient who consulted the doctor yesterday returned home satisfied.
(الأطباء) المريض الذي استشار الطبيب بالأمس عاد الى المنزل مطمئنا.

B.44. The teacher who met the writer happily likes reading about literature.
(الأدباء) المعلم الذي استقبل الأديب بسعادةٍ يحب القراءة عن الأدب.

B.45. The representative who talked to the Khalif yesterday works hard to get a promotion.
(الخلفاء) النائب الذي كلم الخليفة بالأمس يجتهد في العمل للحصول على ترقية.

B.46. The president who hurriedly called the ally tries to reign the situation.
(الحلفاء) الرئيس الذي استدعى الحليف باستعجال يحاول التحكم بزمام الأمور.

B.47. The manager who phoned the partner in the morning plans to expand the branches of the company.
(الشركاء) المدير الذي هاتف الشريك بالصباح يخطط لتوسيع فروع الشركة.

B.48. The chef who invited the guest in the evening masters preparing various delicious dishes.
(النزلاء) الطاهي الذي دعا النزيل بالمساء يتقن إعداد أصناف لذيذة من الطعام.

3 Complete Materials — Experiment 4

C.1. The nurse who treated the patient (fem.) carefully studies at the university hospital.
الممرضة التي عالجت المريضة بعنايةٍ تدرس في مستشفى الجامعة.

C.2. The queen who looked after the princess recently appears in public every week.
الملكة التي اهتمت بالأميرة حديثاً تظهر في العلن كل أسبوع.

C.3. The seller (fem.) who thanked the customer (fem.) enthusiastically was happy with the large profit.
البائعة التي شكرت الزبونة بحماسٍ فرحت بالفائدة الكبيرة.

C.4. The novelist (fem.) who mentioned the maid of honor accurately sells many books to the public.
الكاتبة التي ذكرت الوصيفة بدقةٍ تبيع كتبا كثيرة للجمهور.

C.5. The coach (fem.) who was very interested in the player (fem.) worked at the National Fencing Academy.
المدربة التي اهتمت باللاعبة جداً اشتغلت في الأكاديمية الوطنية للمبارزة.

C.6. The midwife who cared for the girl repeatedly volunteers at the university hospital.
القابلة التي اعتنت بالفتاة تكراراً تتطوع في مستشفى الجامعة.

C.7. The soldier (fem.) who met the policewoman yesterday loved the atmosphere at the air base.
الجندية التي قابلت الشرطية بالأمسِ أحبّت العمل في القاعدة الجوية .

C.8. The singer (fem.) who met the dancer (fem.) previously sings with the city orchestra.
المطربة التي استقبلت الراقصة سابقاً تغني مع أوركسترا المدينة.

C.9. The nanny who cared for the schoolgirl affectionately traveled to a new country.
المربية التي ربّت الطالبة بحنانٍ سافرت إلى بلدٍ جديد.

C.10. The producer (fem.) who enthusiastically chose the actress produces a film every month.
المخرجة التي اختارت الممثلة بشغفٍ تنتج فيلما كل شهر.

C.11. The photographer (fem.) who photographed the witch artistically published the photos in a new book.
المصورة التي صورت الساحرة بتفننٍ نشرت الصور في كتاب جديد.

C.12. The maid who helped the nanny earnestly cleans the rooms seven days a week.
الخادمة التي ساعدت المربية بجدٍّ تنظف الغرف سبعة أيام في الأسبوع.

C.13. The announcer (fem.) who invited the historian (fem.) nicely presented a program on TV.
المذيعة التي دعت المؤرّخة بلطفٍ قدمت برنامجاً في التلفاز.

C.14. The model (fem.) who met the accountant (fem.) repeatedly owns a lot of expensive clothes.
العارضة التي التقت بالمحاسِبة تكراراً تملك كثيرا من الملابس الغالية.

C.15. The assistant (fem.) who served the pharmacist (fem.) loyally collected all of the old reports.
المساعِدة التي خدمت الصيدلانيّة بإخلاصٍ جمعت كل التقارير القديمة.

C.16. The journalist (fem.) who interviewed the winner (fem.) by chance writes in many newspapers.
الصحفية التي قابلت الفائزة صدفةٍ تكتب في صحف كثيرة.

C.17. The bedouin (fem.) who visited the farmer (fem.) at night goes to the middle of the desert.
البدوية التي زارت المزارعة ليلاً تذهب إلى وسط الصحراء.

C.18. The doctor (fem.) who treated the girl recently discovered a cure for the terrible disease.
الطبيبة التي عالجت الطفلة مؤخراً اكتشفت شفاء للمرض الرهيب.

C.19. The artist (fem.) who corresponded with the publisher (fem.) eagerly desired a new contract.
الفنانة التي راسلت الناشرة بشغفٍ رغبت في عَقد جديد.

C.20. The student (fem.) who admired the poet (fem.) greatly read many poems last year.
التلميذة التي أُعجبت بالشاعرة بشدةٍ قرأت قصائد كثيرة العام الماضي.

C.21. The director (fem.) who contacted the author (fem.) during the day supervises many large projects.
المديرة التي اتصلت بالمؤلفة نهاراً تشرف على كثيرٍ من المشاريع الكبيرة.

C.22. The dean (fem.) who summoned the professor (fem.) angrily observed a problem in the university departments.
العميدة التي استدعت الأستاذة بغضبٍ لاحظت خللاً في أقسام الجامعة.

C.23. The musician (fem.) who accompanied the singer (fem.) professionally played with the national music group.
الموسيقيّة التي رافقت المغنية بمهنيّةٍ عزفت مع الفرقة الوطنية للموسيقى.

C.24. The ambassador (fem.) who hosted the delegate (fem.) yearly spoke at the United Nations.
السفيرة التي استضافت المندوبة سنوياً تحدثت في الأمم المتحدة.

C.25. The grandmother who met the neighbor (fem.) suddenly talked about the neighborhood issues.
الجدّة التي صادفت الجارة فجأةً تحاورت عن أمور الحي.

C.26. The student (fem.) who met the manager (fem.) yesterday got high grades in the remaining subjects.
الطالبة التي قابلت المديرة البارحة نالت درجات عالية في المواد المتبقية.

C.27. The accountant (fem.) who talked to the employee (fem.) harshly suffered from social problems.
المحاسبة التي حادثت الموظفة بصرامة عانت من مشاكل اجتماعية.

C.28. The study abroad student (fem.) who thanked the official (fem.) a lot studied at one of the best international universities.
المبتعثة التي شكرت المسؤولة بكثرة درست باحدى أرقى الجامعات الدولية.

C.29. The graduate (fem.) who talked to the lecturer (fem.) happily works for extra hours at the library.
الخريجة التي كلمت المحاضِرة بسعادة تعمل ساعات اضافية في المكتبة.

C.30. The painter (fem.) who excitedly interviewed the producer (fem.) painted wonderful paintings
الرسامة التي حاورت المخرجة بإثارة رسمت لوحات فنية رائعة.

C.31. The chef (fem.) who lived next to the trader (fem.) for a long time practices a cooking career skillfully.
الطاهية التي جاورت التاجرة مطولاً تمارس مهنة الطبخ بمهارة.

C.32. The visitor (fem.) who talked to the guide (fem.) in the morning gave a lecture about how to manage time.

الزائرة التي حدثت المرشدة صباحاً القت محاضرة عن كيفية تنظيم الوقت.

C.33. The teacher (fem.) who visited the doctor (fem.) yesterday masters speaking in Arabic and English.

المعلمة التي زارت الطبيبة بالأمس تُجيد التحدث باللغة العربية و الإنجليزية.

C.34. The lawyer (fem.) who accused the guilty woman angrily is trying to find the way to the truth.

المحامية التي اتهمت المذنبة بغضبٍ تحاول الوصول إلى الحقيقة.

C.35. The engineer (fem.) who met the colleague (fem.) daily aspires to get a job at a prominent company.

المهندسة التي قابلت الزميلة يومياً تسعى للحصول على وظيفة في شركة مرموقة.

C.36. The farmer (fem.) who blamed the young lady yesterday loves working at the farm near the park.

المزارعة التي لامت الشابة بالأمسِ تحب العمل في المزرعة المجاورة للحديقة.

C.37. The beginner (fem.) who helped the boss (fem.) in the morning was hired for the military company.

المبتدئة التي ساعدت الرئيسة صباحاً توظفت في الشركة العسكرية.

C.38. The actress who met the announcer (fem.) in the past resigned from the acting career recently.

الممثلة التي قابلت المذيعة بالماضي استقالت من مهنة التمثيل مؤخراً.

C.39. The guard (fem.) who talked to the pupil (fem.) in the morning goes home late every day.

الحارسة التي كلمت التلميذة صباحاً تذهب إلى المنزل في ساعة متاخرة كل يوم.

C.40. The worker (fem.) who gently scolded the maid cares about helping the needy.

العاملة التي وبخت الخادمة برفق تهتم بمساعدة المحتاجين.

C.41. The analyst (fem.) who patiently waited for the reporter (fem.) is trying to educate people about the importance of a clean environment.

المحللة التي انتظرت المراسلة بصبر تسعى لتوعية الشعب على أهمية نظافة البيئة.

C.42. The reporter (fem.) who spoke to the plaintiff (fem.) adeptly interviews the president at international conferences.

المراسلة التي سألت المدعية بنباهة تقابل رئيس الدولة في المؤتمرات العالمية.

C.43. The magician (fem.) who talked to the lady quickly worked at the theater near the village.

الساحرة التي كلمت السيدة بسرعة عملت في المسرح المجاور للقرية.

C.44. The employee (fem.) who accompanied the visitor (fem.) in the morning stays at work until late.

الموظفة التي رافقت الزائرة بالصباح تبقى حتى ساعة متاخرة في العمل.

C.45. The novelist (fem.) who pleasantly shook hands with the designer (fem.) writes international and local novels about literature.

الروائية التي صافحت المصممة بسرور تكتب روايات عالمية و محلية في الادب.

C.46. The researcher (fem.) who calmly called the detective (fem.) provides money for charity society

الباحثة التي هاتفت المحققة بهدوء توفر المال للجمعيات الخيرية.

C.47. The coordinator (fem.) who helped the guard (fem.) devotedly was in the school courtyard.
المنسقة التي عاونت الحارسة بتفان تواجدت في ساحة المدرسة.

C.48. The candidate (fem.) who pleasantly thanked the participant (fem.) took part in the electoral campaign.
المرشحة التي شكرت المشتركة بسرور شاركت في الحملة الانتخابية.

C.49. The judge (fem.) who decisively questioned the thief (fem.) ruled fairly among people.
القاضية التي سألت السارقة بحزم حكمت بالعدل بين الناس.

C.50. The immigrant (fem.) who answered the inspector (fem.) anxiously faced difficulties at the check point.
المهاجرة التي أجابت المفتشة بقلق واجهت صعوبات عند نقطة التفتيش.

C.51. The young girl who helped the grandmother at night works at bakery for sweets.
الفتاة التي ساعدت الجدة بالليل تعمل في مخبز الحلويات.

C.52. The tourist (fem.) who met the driver (fem.) on the road loves traveling to different countries.
السائحة التي صادفت السائقة بالطريق تحب السفر إلى بلدان مختلفة.

C.53. The princess who intelligently answered the journalist (fem.) owns many huge palaces.
الأميرة التي أجابت الصحافية بذكاء تمتلك عدة قصور كبيرة.

C.54. The client (fem.) who consulted the lawyer (fem.) nervously practiced painting for many years.
الموكلة التي استشارت المحامية بتوتر مارست مهنة الرسم لعدة سنوات.

4 Complete Materials — Experiment 5

D.1. The nurse who treated the patient (fem.) carefully studies at the university hospital.
الممرضة التي عالجت المريضة بعنايةٍ تدرس في مستشفى الجامعة.

D.2. The queen who looked after the princess recently appears in public every week.
الملكة التي اهتمت بالأميرة حديثاً تظهر في العلن كل أسبوع.

D.3. The seller (fem.) who thanked the customer (fem.) enthusiastically was happy with the large profit.
البائعة التي شكرت الزبونة بحماسٍ فرحت بالفائدة الكبيرة.

D.4. The novelist (fem.) who mentioned the maid of honor accurately sells many books to the public.
الكاتبة التي ذكرت الوصيفة بدقةٍ تبيع كتبا كثيرة للجمهور.

D.5. The coach (fem.) who was very interested in the player (fem.) worked at the National Fencing Academy.
المدربة التي اهتمت باللاعبة جداً اشتغلت في الأكاديمية الوطنية للمبارزة.

D.6. The obstetrician (fem.) who cared for the child (fem.) repeatedly volunteers at the university hospital.
القابلة التي اعتنت بالطفلة تكراراً تتطوع في مستشفى الجامعة.

D.7. The soldier (fem.) who met the policewoman yesterday loved the atmosphere at the air base.
الجندية التي قابلت الشرطية بالأمسِ أحبّت العمل في القاعدة الجوية.

D.8. The singer (fem.) who met the dancer (fem.) previously sings with the city orchestra.
المطربة التي استقبلت الراقصة سابقاً تغني مع أوركسترا المدينة.

D.9. The prompter (fem.) who cared for the student (fem.) affectionately traveled to a new country.
الملقّنة التي ربّت الطالبة بحنانٍ سافرت إلى بلدٍ جديد.

D.10. The producer (fem.) who enthusiastically chose the actress produces a film every month.
المخرجة التي اختارت الممثلة بشغفٍ تنتج فيلما كل شهر.

D.11. The photographer (fem.) who photographed the magician (fem.) artistically published the photos in a new book.
المصورة التي صورت الساحرة بتفننٍ نشرت الصور في كتاب جديد.

D.12. The maid who helped the sponsor (fem.) earnestly cleans the rooms seven days a week.
الخادمة التي ساعدت الكفيلة بجدٍّ تنظف الغرف سبعة أيام في الأسبوع.

D.13. The announcer (fem.) who invited the historian (fem.) nicely presented a program on TV.
المذيعة التي دعت المؤرّخة بلطفٍ قدمت برنامجاً في التلفاز.

D.14. The translator (fem.) who met the accountant (fem.) repeatedly owns a lot of expensive clothes.
المترجمة التي التقت بالمحاسِبة تكراراً تملك كثيرا من الملابس الغالية.

D.15. The assistant (fem.) who served the pharmacist (fem.) loyally collected all of the old reports.
المساعِدة التي خدمت الصيدلانيّة بإخلاصٍ جمعت كل التقارير القديمة.

D.16. The journalist (fem.) who interviewed the winner (fem.) by chance writes in many newspapers.
الصحفية التي قابلت الفائزة صدفةٍ تكتب في صحف كثيرة.

D.17. The bedouin (fem.) who visited the farmer (fem.) at night goes to the middle of the desert.
البدوية التي زارت المزارعة ليلاً تذهب إلى وسط الصحراء.

D.18. The doctor (fem.) who treated the girl recently discovered a cure for the terrible disease.
الطبيبة التي عالجت الصبية مؤخراً اكتشفت شفاء للمرض الرهيب.

D.19. The artist (fem.) who corresponded with the publisher (fem.) eagerly desired a new contract.
الفنانة التي راسلت الناشرة بشغفٍ رغبت في عَقد جديد.

D.20. The student (fem.) who admired the poet (fem.) greatly read many poems last year.
التلميذة التي أُعجبت بالشاعرة بشدةٍ قرأت قصائد كثيرة العام الماضي.

D.21. The director (fem.) who contacted the author (fem.) during the day supervises many large projects.
المديرة التي اتصلت بالمؤلفة نهاراً تشرف على كثيرٍ من المشاريع الكبيرة.

D.22. The dean (fem.) who summoned the professor (fem.) angrily observed a problem in the university departments.
العميدة التي استدعت الأستاذة بغضبٍ لاحظت خللاً في أقسام الجامعة.

D.23. The musician (fem.) who accompanied the singer (fem.) professionally played with the national music group.
الموسيقيّة التي رافقت المغنية بمهنيّةٍ عزفت مع الفرقة الوطنية للموسيقى.

D.24. The ambassador (fem.) who hosted the delegate (fem.) yearly spoke at the United Nations.
السفيرة التي استضافت المندوبة سنوياً تحدثت في الأمم المتحدة.

D.25. The grandmother who met the neighbor (fem.) suddenly talked about the neighborhood issues.
الجدّة التي صادفت الجارة فجأةً تحاورت عن أمور الحي.

D.26. The student (fem.) who met the manager (fem.) yesterday got high grades in the remaining subjects.
الطالبة التي قابلت المديرة البارحة نالت درجات عالية في المواد المتبقية.

D.27. The accountant (fem.) who talked to the employee (fem.) harshly suffered from social problems.
المحاسبة التي حادثت الموظفة بصرامة عانت من مشاكل اجتماعية.

D.28. The volunteer (fem.) who thanked the official (fem.) a lot studied at one of the best international universities.
المتطوعة التي شكرت المسؤولة بكثرة درست باحدى أرقى الجامعات الدولية.

D.29. The graduate (fem.) who talked to the lecturer (fem.) happily works for extra hours at the library.
الخريجة التي كلمت المحاضِرة بسعادة تعمل ساعات اضافية في المكتبة.

D.30. The painter (fem.) who excitedly interviewed the producer (fem.) painted wonderful paintings
الرسامة التي حاورت المخرجة بإثارة رسمت لوحات فنية رائعة.

D.31. The chef (fem.) who lived next to the trader (fem.) for a long time practices a cooking career skillfully.
الطاهية التي جاورت التاجرة مطولاً تمارس مهنة الطبخ بمهارة.

D.32. The visitor (fem.) who talked to the guide (fem.) in the morning gave a lecture about how to manage time.
الزائرة التي حدثت المرشدة صباحاً القت محاضرة عن كيفية تنظيم الوقت.

D.33. The teacher (fem.) who visited the doctor (fem.) yesterday masters speaking in Arabic and English.
المعلمة التي زارت الطبيبة بالأمس تُجيد التحدث باللغة العربية و الإنجليزية.

D.34. The lawyer (fem.) who accused the guilty woman angrily is trying to find the way to the truth.
المحامية التي اتهمت المذنبة بغضبٍ تحاول الوصول إلى الحقيقة.

D.35. The engineer (fem.) who met the colleague (fem.) daily aspires to get a job at a prominent company.
المهندسة التي قابلت الزميلة يومياً تسعى للحصول على وظيفة في شركة مرموقة.

D.36. The farmer (fem.) who blamed the young person (fem.) yesterday loves working at the farm near the park.
المزارعة التي لامت الشابة بالأمسِ تحبّ العمل في المزرعة المجاورة للحديقة.

D.37. The beginner (fem.) who helped the boss (fem.) in the morning was hired for the military company.
المبتدئة التي ساعدت الرئيسة صباحاً توظفت في الشركة العسكرية.

D.38. The actress who met the announcer (fem.) in the past resigned from the acting career recently.
الممثلة التي قابلت المذيعة بالماضي استقالت من مهنة التمثيل مؤخراً.

D.39. The guard (fem.) who talked to the pupil (fem.) in the morning goes home late every day.
الحارسة التي كلمت التلميذة صباحاً تذهب إلى المنزل في ساعة متاخرة كل يوم.

D.40. The worker (fem.) who gently scolded the maid cares about helping the needy.
العاملة التي وبخت الخادمة برفق تهتم بمساعدة المحتاجين.

D.41. The analyst (fem.) who patiently waited for the reporter (fem.) is trying to educate people about the importance of a clean environment.
المحللة التي انتظرت المراسلة بصبر تسعى لتوعية الشعب على أهمية نظافة البيئة.

D.42. The reporter (fem.) who spoke to the plaintiff (fem.) adeptly interviews the president at international conferences.
المراسلة التي سألت المدعية بنباهة تقابل رئيس الدولة في المؤتمرات العالمية.

D.43. The magician (fem.) who talked to the lady quickly worked at the theater near the village.
الساحرة التي كلمت السيدة بسرعة عملت في المسرح المجاور للقرية.

D.44. The employee (fem.) who accompanied the visitor (fem.) in the morning stays at work until late.
الموظفة التي رافقت الزائرة بالصباح تبقى حتى ساعة متاخرة في العمل.

D.45. The novelist (fem.) who pleasantly shook hands with the designer (fem.) writes international and local novels about literature.
الروائية التي صافحت المصممة بسرور تكتب روايات عالمية و محلية في الادب.

D.46. The researcher (fem.) who calmly called the detective (fem.) provides money for charity society
الباحثة التي هاتفت المحققة بهدوء توفر المال للجمعيات الخيرية.

D.47. The coordinator (fem.) who helped the guard (fem.) devotedly was in the school courtyard.

المنسقة التي عاونت الحارسة بتفانٍ تواجدت في ساحة المدرسة.

D.48. The candidate (fem.) who pleasantly thanked the participant (fem.) took part in the electoral campaign.

المرشحة التي شكرت المشتركة بسرور شاركت في الحملة الانتخابية.

D.49. The judge (fem.) who decisively questioned the thief (fem.) ruled fairly among people.

القاضية التي سألت السارقة بحزم حكمت بالعدل بين الناس.

D.50. The immigrant (fem.) who answered the inspector (fem.) anxiously faced difficulties at the check point.

المهاجرة التي أجابت المفتشة بقلق واجهت صعوبات عند نقطة التفتيش.

D.51. The pharmacist (fem.) who helped the grandmother at night works at bakery for sweets.

الصيدلانيّة التي ساعدت الجّدة بالليل تعمل في مخبز الحلويات.

D.52. The tourist (fem.) who met the driver (fem.) on the road loves traveling to different countries.

السائحة التي صادفت السائقة بالطريق تحبّ السفر إلى بلدان مختلفة.

D.53. The princess who intelligently answered the journalist (fem.) owns many huge palaces.

الأميرة التي أجابت الصحافية بذكاء تمتلك عدة قصور كبيرة.

D.54. The client (fem.) who consulted the lawyer (fem.) nervously practiced painting for a few years.

الموكلة التي استشارت المحامية بتوتر مارست مهنة الرسم لعدة سنوات.
